# IGF2BP1 promotes mesenchymal cell properties and migration of tumor-derived cells by enhancing the expression of LEF1 and SNAI2 (SLUG)

**DOI:** 10.1093/nar/gkt410

**Published:** 2013-05-15

**Authors:** Anne Zirkel, Marcell Lederer, Nadine Stöhr, Nikolaos Pazaitis, Stefan Hüttelmaier

**Affiliations:** Institute of Molecular Medicine, Department of Molecular Cell Biology, Martin-Luther-University, Heinrich-Damerow-Str.1, 06120 Halle, Germany

## Abstract

The oncofetal IGF2 mRNA-binding protein 1 (IGF2BP1) controls the migration and invasiveness of primary as well as tumor-derived cells *in vitro*. Whether the protein also modulates epithelial-mesenchymal-transition (EMT), a hallmark of tumor progression involved in tumor cell dissemination, remained elusive. In this study, we reveal that IGF2BP1 enhances mesenchymal-like cell properties in tumor-derived cells by promoting the expression of the transcriptional regulators LEF1 and SLUG (SNAI2). IGF2BP1 associates with LEF1 transcripts and prevents their degradation in a 3′-UTR-dependent manner resulting in an upregulation of LEF1 expression. LEF1 promotes transcription of the mesenchymal marker fibronectin by associating with the fibronectin 1 promoter. Moreover, LEF1 enforces the synthesis of the ‘EMT-driving’ transcriptional regulator SNAI2. Accordingly, IGF2BP1 knockdown causes MET-like (mesenchymal-epithelial-transition) morphological changes, enhances the formation of cell–cell contacts and reduces cell migration in various mesenchymal-like tumor-derived cells. However, in epithelial-like tumor-derived cells characterized by a lack or low abundance of IGF2BP1, the protein fails to induce EMT. These findings identify IGF2BP1 as a pro-mesenchymal post-transcriptional determinant, which sustains the synthesis of ‘EMT-driving’ transcriptional regulators, mesenchymal markers and enhances tumor cell motility. This supports previous reports, suggesting a role of IGF2BP1 in tumor cell dissemination.

## INTRODUCTION

Epithelial-mesenchymal-transition (EMT) is essential during embryogenesis and is considered a hallmark in the progression of carcinomas [reviewed in (1,2)]. In cancer, the term EMT describes a complex network of molecular and cellular trans-differentiation phenomena by which epithelial-like tumor cells acquire mesenchymal-like properties leading to reduced inter-cellular adhesion, increased migratory capacity and elevated invasive potential. Accumulating evidence indicates that the post-transcriptional control of gene expression facilitated by microRNAs essentially modulates EMT and its reversal, mesenchymal-epithelial-transition (MET). One of the most studied post-transcriptional mechanisms promoting EMT is facilitated via the miR-200 family. This antagonizes TGF-β (TGFB)-induced EMT by interfering with the expression of ZEBs, two key transcriptional repressors of E-cadherin (CDH1) [reviewed in (3)]. Another double-negative feedback loop modulating cell plasticity essentially relies on the miR-34 family, which links p53 signaling and negative regulation of Snail (SNAI1) expression, another ‘EMT-driving’ transcriptional regulator (4,5). Surprisingly, little is known about the role of RNA-binding proteins (RBPs) in modulating EMT in cancer-derived cells, although at least two RBPs were proposed as essential modulators of malignant EMT/MET. The splicing regulator Sam68 was shown to control EMT through alternative splicing-activated nonsense-mediated mRNA decay of the proto-oncogene SF2/ASF (6). More recently, it was demonstrated that La enhances the IRES-mediated translation of the extracellular matrix protein laminin B1 during malignant EMT (7).

IGF2 mRNA-binding proteins (IGF2BPs) comprise a group of three proteins, two of which, IGF2BP1 and IGF2BP3, were proposed to serve essential functions during embryogenesis and in cancer [reviewed in (8–10)]. In contrast to IGF2BP2, which appears to be the main or even exclusive IGF2BP member expressed in non-neoplastic adult tissue, IGF2BP1 and IGF2BP3 were found to be severely upregulated in various cancers [reviewed in (8,11)]. However, in view of the multitude of descriptive studies indicating elevated expression of IGF2BP1/3 to correlate with tumor aggressiveness, their role in cancer cells remains poorly understood. Studies in tumor-derived as well as primary cells suggest two main functions of IGF2BPs, growth control and the regulation of cell migration. Evidence for a role in regulating cell growth and proliferation was provided by findings indicating that all IGF2BPs promote or interfere with IGF2 protein synthesis. IGF2BP1 was suggested to inhibit IGF2 mRNA translation by associating with the highly structured leader3 5′-UTR of the transcript, one of four alternative 5′-UTRs reported (12,13). In contrast, IGF2BP2 and IGF2BP3 were shown to enhance translation of the IGF2 mRNA, presumably involving the phosphorylation of IGF2BP2 by mTORC1 (14,15). Another target transcript via which IGF2BP1 was proposed to modulate cell proliferation is the oncogenic transcriptional regulator c-Myc (MYC). On associating with a rare-codon comprising coding region determinant (CRD), IGF2BP1 was shown to prevent cleavage of the MYC mRNA by endonucleases when ribosomes are slowed down on entering the rare-codon region of the CRD (16). In agreement, we observed that IGF2BP1 knockdown results in a significantly reduced half-life of the MYC mRNA in most tumor-derived cells and accordingly is associated with severely decreased cell proliferation (17,18). Finally, it was proposed that IGF2BP1 modulates β-catenin (CTNNB1) signaling (19,20). On the one hand, it was shown that IGF2BP1 transcription is enhanced in a CTNNB1/TCF-dependent manner but then negatively feeds back on CTNNB1-dependent signaling by enhancing the expression of beta-TrCP1, which among others facilitates CTNNB1 protein degradation (21). On the contrary, IGF2BP1 was demonstrated to enhance CTNNB1 expression by preventing degradation of the CTNNB1 mRNA (20). The interplay of IGF2BP1 and CTNNB1-dependent signaling was moreover suggested to negatively regulate the migration of breast cancer-derived cells *in vitro* (22). In contrast, various observations indicate that IGF2BP1 and its ortholog Vg1RBP/Vera promote the migration of primary as well as tumor-derived cells [reviewed in (23)]. In *Xenopus*, Vg1RBP/Vera was shown to enhance the migration of neural crest cells during development (24). In agreement, we reported that IGF2BP1 promotes the directed migration of tumor cells derived from osteosarcoma, ovarian carcinoma as well as glioblastoma (25). This was also demonstrated in colorectal as well as mammary carcinoma-derived cells, in which IGF2BPs enhance the formation of lamellipodia and promote directed migration, respectively (26,27). Finally, IGF2BP1 and IGF2BP3 were suggested to enhance the invasive potential of cervical carcinoma-derived HeLa cells by interfering with the degradation of the CD44 mRNA (28). This results in elevated CD44 expression and enforced formation of invadopodia *in vitro*. One common theme in the IGF2BP-facilitated regulation of cell migration, adhesion and potentially invasion is the regulation of actin dynamics [reviewed in (23)]. IGF2BP1, also termed zipcode-binding protein (ZBP) in chicken, facilitates the localization of β-actin (ACTB) encoding transcripts to the leading edge of primary fibroblasts as well as the growth cones of developing neurons (29–31). This enforcement of spatially restricted ACTB mRNA levels was proposed to provide a pool of transcripts for the rapid activation of ACTB protein synthesis and thus enhance directed cell protrusion in response to external guidance cues [reviewed in (32)]. In agreement, IGF2BP1 was observed to promote actin-driven neurite protrusion by controlling translation of the ACTB mRNA in a spatiotemporal and Src-kinase controlled manner (33). Moreover, IGF2BP1 and its ortholog Vg1RBP/Vera were revealed to modulate growth cone guidance during neuronal development (34,35). Like in primary neurons, IGF2BP1 also serves essential roles in regulating actin dynamics in tumor-derived cells. In recent studies, we proposed that the protein modulates the cellular G-/F-actin equilibrium by controlling ACTB protein synthesis and HSP27 (HSPB1)-dependent sequestering of monomeric actin (25). The latter is facilitated by IGF2BP1-directed inhibition of MAPK4 mRNA translation, which limits the activation of MK5-directed phosphorylation of HSPB1 and thereby reduces sequestering of monomeric actin by this small heat shock protein [reviewed in (23)]. Despite these various studies indicating a regulatory role of IGF2BPs, in particular IGF2BP1, in directing the migration and invasive potential of tumor-derived cells *in vitro*, it remains elusive whether IGF2BPs also regulate tumor cell dissemination *in vivo*. One key aspect that remains to be addressed in this respect is whether IGF2BPs serve a role in modulating mesenchymal versus epithelial properties of cancer-derived cells. This has been barely investigated, although one recent study suggests that IGF2BP1 promotes the formation of cell–cell contacts by enhancing the spatially restricted expression of CDH1 in proximity to cell–cell contacts (36).

## MATERIALS AND METHODS

### Plasmids

Full-length LEF1 (NM_016269) as well as alternative 3′-UTRs of LEF1 isoforms (A: NM_016269; NM_001130713; NM_001166119; B: NM_001130714) were generated by RT-PCR from HEK293 cells. The LEF1 coding sequence was inserted via BamHI/ EcoRI in pcDNA3.1zeo-Flag, pLVX-puro and pLVX-puro GFP plasmids, respectively. The LEF1 3′UTRs were inserted via EcoRI/ XhoI into pcDNA3.1neo-LUC (LUC: Firefly luciferase), as recently described (25). The SNAI2 3′UTR was amplified by RT-PCR from HT-144 cells and inserted into the pmirGLO (Promega) vector via BamHI/ XhoI. The fibronectin (FN1) minimal promoter (−839 to +1), the 5′UTR (+1 to +266_ATG) and the starting ATG of human FN1 (Chr.2q34) were identified by *in silico* prediction using Proscan (http://www-bimas.cit.nih.gov/molbio/proscan/). The longest FN1-promoter fragment (1105 nt; −839 to starting ATG) as well as SNAI2 promoter (37) was PCR-amplified from HEK293 genomic DNA and transferred into pGL4.21 (Promega) via XhoI/ BglII sites. ShRNA-encoding lentiviral vectors were generated by inserting annealed oligonucleotides via BamHI/EcoRI in the modified pLVX-shRNA2 or modified pLVX-shRNA2-Crimson-puro. In the latter, the ZsGreen cassette was replaced by a PGK-promoter driven cassette encoding for E2-Crimson fused to the puromycin resistance via an EMCV IRES to allow for tracing and selection of transduced cells. All PCR-amplified products and modified vectors were validated by sequencing. The following plasmids were obtained from Addgene: SNAI2-directed shRNA lentiviral vector (ID: 10905); SNAI1 cDNA (ID: 36976), subcloned in pLVX-puro GFP; SNAI2 cDNA (ID: 36986), subcloned in pLVX-puro GFP; SNAI1 promoter (ID: 31694). For PCR primers used for cloning and plasmids, refer to Supplementary Table ST1.

### Cell culture, transfection and lentiviruses

All cells were cultured in Dulbecco’s modified Eagle’s medium supplemented with 10% fetal bovine serum. To reduce bias by cell density-dependent regulation of epithelial or mesenchymal marker expression, cells were harvested or analyzed at ∼80% confluence. Cells were transfected with siRNAs by RNAiMax (72 h) or plasmids by Lipofectamine 2000 (48 h), as previously described (25). SiRNA and shRNA sequences are listed in Supplementary Tables ST1 and ST2. For knockdown-recovery studies, cells were co-transfected with indicated shRNA encoding and Flag-tagged protein-encoding plasmids for 72 h. Where indicated, cells were treated with actinomycin D (ActD; 5 µM) to block mRNA synthesis and monitor mRNA decay, as recently described (25). Lentiviruses were produced essentially as recently described (25). Transduced cell populations were subsequently cultured in the presence of puromycin (1 µg/ml). All lentiviral transfer vectors are indicated in Supplementary Table ST1.

### Immunofluorescence and microscopy

Cells were grown on coverslips (48 h) and processed for immunostaining with indicated antibodies on fixation by formaldehyde, as previously described (38). Nuclei were stained by DAPI, and F-actin was labeled by phalloidin-TRITC. Representative images are shown. Images were acquired using a Leica LSM-SP5× microscope, as recently described (25). Antibodies used for immunostaining are indicated in Supplementary Table ST3. Bright field images of living cells were acquired using a Nikon TE-100 inverse microscope equipped with a Nikon CoolPix990 camera and a 40× Plan Apo objective. For wound closure analyses, cells (1 × 10^5^/well) were cultured for 24 h in a 24-well plate and scratched before time lapse microscopy using a Leica LSM-SP5× microscope equipped with a Ludin Cube live cell chamber and a 20× Plan Fluor objective. Images were acquired every 15 min. Movies of all cell populations were analyzed simultaneously using automated cell segmentation and wound closure algorithms recently described (39).

### RT-PCR and qRT-PCR

RNA isolation and reverse transcription were carried out as previously described (25). Briefly, total RNA was isolated by Trizol reagent followed by Chloroform extraction. Reverse transcription was performed using M-MLV-RT (Promega) and oligo-dT priming at 42°C for 2 h. The cDNA samples were then analyzed using SYBR® Select Master Mix (Life Technologies) and the 7900HT Fast Real-Time PCR System (Applied Biosystems) in triplicates. RNA abundance was determined using the ΔC_t_ or ΔΔC_t_ method, respectively. Primers used for quantitative PCR analyses are listed in Supplementary Table ST4.

### Luciferase reporter analysis

Luciferase activities were determined using DualGlo reagent (Promega), as previously reported (18,25). For promoter analyses, HEK293 cells were co-transfected with indicated luciferase reporters and protein encoding plasmids for 30 h or shRNA encoding vectors for 48 h. For analyses of 3′UTR-containing reporters, cells were transfected with siRNAs 48 h before the transfection of luciferase reporters for an additional 24 h. Renilla luciferase served as an internal normalization control in all analyses.

### Western blotting

For western blotting, cells were harvested by a rubber policeman to minimize degradation of trans-membrane proteins like CDH1. Total protein was extracted in RIPA-buffer [20 mM Tris–HCl (pH 7.5), 150 mM NaCl, 1 mM EDTA, 1 mM EGTA, 1% NP-40, 1% sodium deoxycholate , 2.5 mM sodium pyrophosphate, 1 mM beta-glycerophosphate, 1 mM Na_3_VO_4_] supplemented with protease inhibitor cocktail (Sigma Aldrich). Protein abundance was analyzed by western blotting with indicated antibodies using the Odyssey infrared scanner (LICOR), as previously described (18,25). Antibodies used for western blotting are indicated in Supplementary Table ST3.

### Flow cytometry

The volume and number of detached cells was determined by flow cytometry measurements using a MACSQuant (Miltenyi Biotec). The relative cell size was determined by forward scattering.

### Enzyme-linked immunosorbent assay

Soluble FN1 protein levels secreted by HEK293 cells were determined using a human FN1 enzyme-linked immunosorbent assay (ELISA) (Boster Biological Technology). The assay was performed according to the manufacturer’s instructions. HEK293 cells were transfected with the indicated siRNAs for 72 h and starved with fetal bovine serum free Dulbecco’s modified Eagle’s medium for 16 h before the collection of the cell culture supernatant. Fibronectin protein amounts were normalized to cell numbers determined by flow cytometry.

### RNA-immunoprecipitation

HEK293 cells were harvested and cross-linked with 0.1% formaldehyde in PBS (10^7^ cells in 1 mL) for 10 min before quenching by 0.1 M Tris–HCl for 5 min. Cells were extracted in RNA-immunoprecipitation (RIP)-buffer [10 mM HEPES (pH 7.2), 150 mM KCl, 5 mM MgCl_2_, 0.5% NP40] supplemented with protease inhibitor cocktail (Sigma Aldrich) and RNasin (Promega). Antibodies for control-IP [immunoglobulin G (IgG) mouse] or the IGF2BP1-IP were coupled to proteinG Dynabeads (Life Technologies) in wash buffer [WB: 50 mM Tris–HCl (pH 7.4), 300 mM NaCl, 0.01% NP40, 5 mM MgCl_2_] supplemented with yeast tRNA (20 µg/ml). After antibody coupling to beads, cell lysates were added in a 1:1 (v/v) ratio and incubated at 4°C overnight with constant agitation. The beads were washed once with WB and three times with WB containing 0.5 M urea. Protein–RNA complexes were eluted in WB supplemented with 1% SDS at 65°C for 10 min. Reversal of the cross-link was achieved by adding proteinase K (Roche) for 1 h at 65°C. RNA was purified by phenol-chloroform extraction. RNA samples were treated with RQ1-DNase before reverse transcription with M-MLV reverse transcriptase and random hexamer primers. RNA abundance was assessed by semi-quantitative and quantitative PCR using primers listed in Supplementary Table ST4.

### Chromatin immunoprecipitation

The chromatin immunoprecipitation was performed using the SimpleChIP™ Enzymatic Chromatin IP Kit (Cell Signaling) essentially according to the manufacturer’s instructions. For each ChIP experiment, 4 × 10^7^ HEK293 cells were used. Co-purification of indicated genomic DNA fragments was analyzed by semi-quantitative as well as quantitative PCR using primers listed in Supplementary Table ST4.

## RESULTS

### IGF2BP1 promotes mesenchymal cell properties

**Figure 1.**
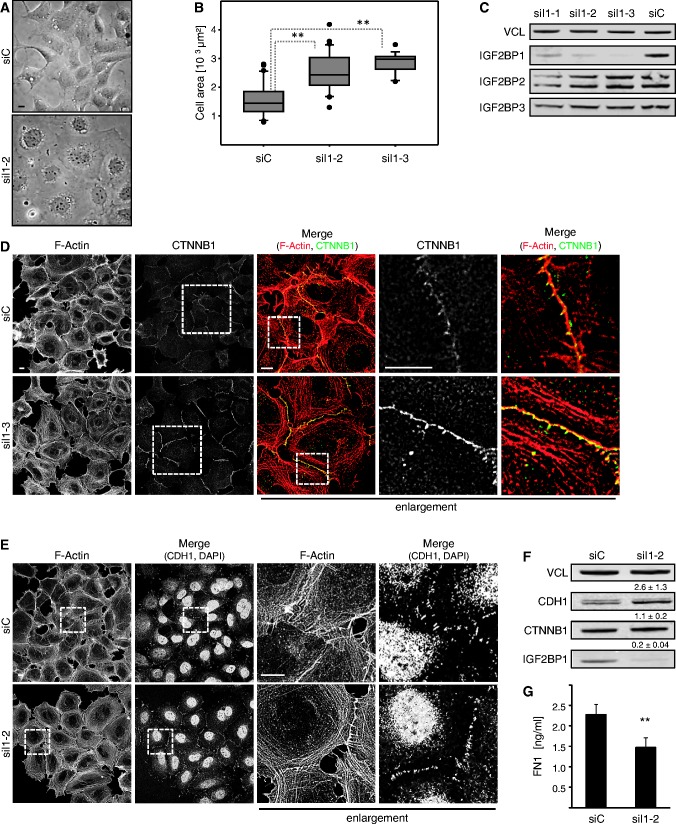
IGF2BP1 knockdown promotes epithelial-like cell properties in HEK293 cells. (**A** and **B**) HEK293 cells were transfected with control (siC) or IGF2BP1-directed (siI1-2 or siI1-3) siRNAs for 72 h. Cell morphology was monitored by light microscopy (A). The size of adherent cells was analyzed on immunostaining for CTNNB1 as well as F-actin labeling by phalloidin and is depicted as box plots (B). Images were acquired by LSM microscopy. Adherent cells were traced by manual labeling using CTNNB1-defined cell borders to determine the cell area (µm^2^) using the Leica-SP5× software (also see Supplementary Figure S1A). (**C**) HEK293 cells were transfected with control (siC) or three distinct IGF2BP1-directed siRNAs (siI1-1, siI1-2 or siI1-3) for 72 h. IGF2BP1 paralogue-specific knockdown was analyzed by western blotting using IGF2BP1-, IGF2BP2- or IGF2BP3-directed monoclonal antibodies. VCL served as a loading control. (**D** and **E**) HEK293 cells were transfected with indicated siRNAs as in (A). The F-actin cytoskeleton and cell–cell contact formation was analyzed by phalloidin labeling and immunostaining for CTNNB1 (D) or CDH1 (E). Where indicated nuclei were stained by DAPI. Enlargements of boxed regions (left panels) are shown in the right panels (enlargement). Note the enrichment of CTNNB1 and CDH1 at adherens junctions and a knockdown-induced enhancement of cortical F-Actin (also see Supplementary Figure S1F). Representative images were acquired by LSM microscopy; bars, 10 µm. (**F**) HEK293 cells were transfected with indicated siRNAs as in (A). CDH1, CTNNB1 and IGF2BP1 protein abundance was analyzed by western blotting with indicated antibodies. Protein levels on IGF2BP1 knockdown were determined relative to controls (siC) by normalization to VCL, as indicated above panels. Representative western blots of three independent analyses are shown. (**G**) Soluble FN1 levels were analyzed by ELISA in HEK293 cells transfected with indicated siRNAs for 72 h. Statistical significance was validated by Student’s *t*-test: **P < 0.005. Error bars indicate standard deviation (SD) of at least three independent analyses.

Aiming to reveal whether IGF2BP1 modulates mesenchymal versus epithelial cell properties, we analyzed its role in transformed embryonic kidney-derived 293 A (HEK293) cells. HEK293 cells express all IGF2BPs, in particular substantial amounts of IGF2BP1, and show epithelial-like as well as mesenchymal-like cell characteristics with few CTNNB1/CDH1-positive cell–cell contacts and expression of mesenchymal markers like FN1. The transient knockdown of IGF2BP1 induced an increased size and apparent flattening of adherent HEK293 cells (Figure 1A). The observed enlargement of adhesive cells was confirmed by the quantitative assessment of cell size using LSM-microscopy and two distinct IGF2BP1-directed siRNAs (Figure 1B and Supplementary Figure S1A). Flow cytometry revealed that the overall size of detached cells, as determined by forward scattering, remained largely unaffected by IGF2BP1 knockdown (Supplementary Figure S1B). This suggested that the shift in cell size was due to altered cytoskeletal organization rather than an overall increase in cell mass. Notably, the used siRNAs were highly IGF2BP paralogue selective supporting an IGF2BP1-dependent role in controlling cell morphology (Figure 1C and Supplementary Figure S1C). Consistent with the inhibitory role of IGF2BP1 in ACTB mRNA translation (25,33), depletion of the protein resulted in an increase of ACTB protein levels in HEK293 cells (Supplementary Figure S1D). To evaluate whether IGF2BP1 depletion also enhanced epithelial-like cell characteristics, the formation of cell–cell contacts was analyzed by immunostaining for CTNNB1 and CDH1 as well as monitoring F-actin organization by phalloidin (Figure 1D, E and Supplementary Figure S1F). In contrast to the previously observed disturbance of stress-fibers in U2OS cells (25), IGF2BP1 knockdown induced an enrichment of cortical actin in HEK293 cells. Concomitantly, the recruitment of CTNNB1 as well as CDH1 to cell–cell contacts sites was markedly pronounced by IGF2BP1 knockdown using two distinct siRNAs. This morphological re-organization was associated with a modest increase in CDH1 protein and mRNA levels (Figure 1F and Supplementary Figure S1E). CTNNB1 protein amounts remained largely unaffected, although CTNNB1 mRNA abundance was decreased by IGF2BP1 knockdown, as previously described (20). On the contrary, the abundance of secreted FN1 protein as well as FN1 mRNA was significantly decreased by IGF2BP1 depletion (Figure 1G and Supplementary Figure S1E). Taken together, these observations suggested that IGF2BP1 depletion promotes epithelial-like and interferes with mesenchymal-like cell properties.

### IGF2BP1 promotes the expression of LEF1

In recent studies, we identified various novel candidate target transcripts of IGF2BP1 using the selective stabilization of mRNAs by the protein during cellular stress as a screening criterion in osteosarcoma-derived U2OS cells (25). These analyses suggested LEF1 paralogue encoding mRNAs as putative target transcripts of IGF2BP1. Notably, LEF1 was identified as a regulator of FN1 and CDH1 expression in tumor-derived cells and was proposed to act in both, a CTNNB1-dependent as well as -independent manner (40,41). Hence, we hypothesized that IGF2BP1-directed regulation of pro-mesenchymal cell properties could be facilitated at least in part via LEF1.

**Figure 2.**
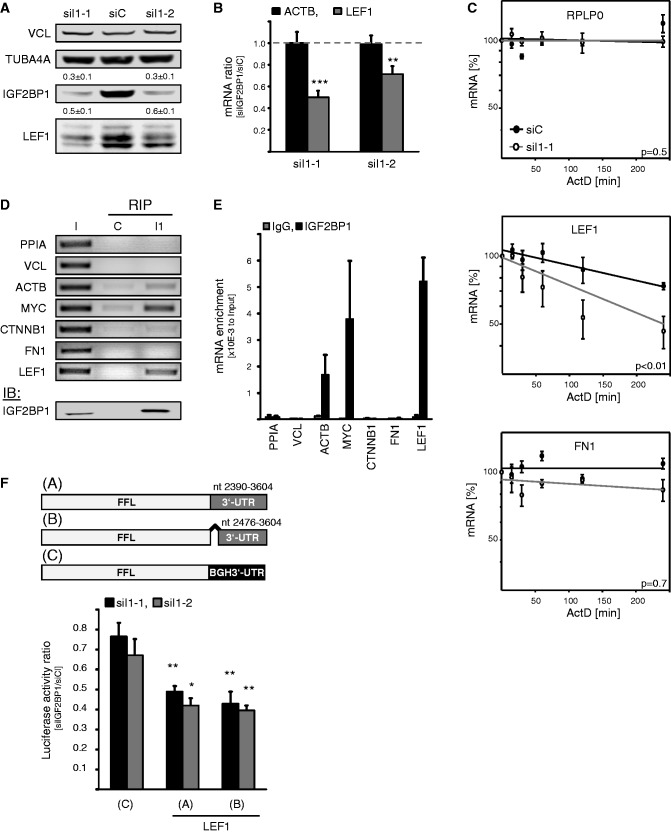
IGF2BP1 promotes LEF1 expression by preventing LEF1 mRNA degradation. (**A** and **B**) HEK293 cells were transfected with control (siC) or indicated IGF2BP1-directed (siI1-1, siI1-2) siRNAs for 72 h. Protein abundance on IGF2BP1 knockdown was determined relative to controls (siC) by western blotting using VCL and TUBA4A for cross-normalization, as indicated above panels. Representative western blots of three independent analyses are shown. ACTB and LEF1 mRNA levels were analyzed by qRT-PCR. Changes in RNA abundance on IGF2BP1 knockdown (siIGF2BP1) were determined relative to controls (siC) by the ΔΔC_t_-method using PPIA for normalization. (**C**) RNA decay was monitored in HEK293 cells transfected with indicated siRNAs for 72 h by blocking mRNA synthesis using ActD (5 µM) for indicated times. RNA levels were determined by qRT-PCR using normalization to PPIA by the ΔΔC_t_-method. RPLP0 served as a control. RNA decay is depicted in semi-logarithmic scale. Statistical significance determined over three independent analyses was analyzed by Student’s *t*-test, as shown in panels (*P*-values). (**D** and **E**) The association of indicated mRNAs with IGF2BP1 in HEK293 cells was analyzed by RIP using formaldehyde fixation to stabilize mRNPs prior purification. Endogenous IGF2BP1 was immunopurified (I1) by a monoclonal antibody, as indicated by western blotting in the lower panel (IB). Co-purification of indicated mRNAs was analyzed relative to the input fraction (I, 10% of cell lysates) by semi-quantitative (D) as well as qRT-PCR (E). IgG-agarose served as a control (C) for unspecific mRNA binding. The enrichment of mRNAs by immunopurification of IGF2BP1 (I1) was determined relative to the input fraction by using the ΔC_t_-method (E). (**F**) Upper panel: Scheme of used Firefly reporters comprising the two alternative LEF1 3′-UTRs (A: Acc.No., NM_016269 /001130713/ 001166119; B: Acc.No., NM_001130714) or the vector-encoded BGH-3′UTR (C). Lower panel: HEK293 cells were transfected with control or indicated IGF2BP1-directed siRNAs for 48 h before the co-transfection of Firefly luciferase reporters (A–C: see scheme in upper panel) and Renilla luciferase control reporters for 24 h. Changes in Firefly luciferase reporter activities on IGF2BP1 knockdown (siIGF2BP1) were determined relative to controls (siC) on normalization by Renilla activities. Statistical significance was validated by Student’s *t*-test: *P < 0.05; **P < 0.005; ***P < 0.0005. Error bars indicate SD of at least three independent analyses.

The knockdown of IGF2BP1 with two distinct siRNAs induced a significant decrease in the levels of all three major LEF1 protein isoforms observed in HEK293 cells (Figure 2A). This was well correlated with a modest, but significant, decrease in LEF1 mRNA levels at steady state, whereas ACTB transcript abundance remained largely unaffected, as previously demonstrated (Figure 2B). Notably, the steady state levels of LEF1 mRNAs were not affected by the knockdown of IGF2BP2 or IGF2BP3 (Supplementary Figure S2A and B). This suggested that IGF2BP1 promoted LEF1 expression by stabilizing the LEF1 mRNA, as previously shown for CD44, MYC or PTEN (18,25,28). To evaluate a role of IGF2BP1 in preventing LEF1 mRNA degradation, the turnover of LEF1 transcripts was monitored on IGF2BP1 knockdown by using ActD to block mRNA synthesis. These analyses revealed a significant destabilization of LEF1 mRNAs in response to IGF2BP1 depletion, whereas RPLP0 as well as FN1 mRNA turnover remained largely unaffected (Figure 2C). This suggested that the IGF2BP1 knockdown induced decrease in FN1 resulted from an indirect impairment of FN1 transcription. In contrast, IGF2BP1 interfered with LEF1 mRNA turnover, presumably by associating with LEF1 transcripts. The latter was tested by RIP using formaldehyde-facilitated cross-linking to stabilize cytoplasmic mRNPs followed by IGF2BP1 immunopurification (Figure 2D and E). Semi- as well as quantitative RT-PCR confirmed MYC and ACTB mRNAs as direct targets of IGF2BP1, as previously demonstrated in U2OS cells (25). Selective association was also observed for LEF1 transcripts, whereas no association was determined for PPIA, vinculin (VCL) and FN1-encoding mRNAs, indicating LEF1, but not N1, as a direct target transcript of IGF2BP1. Surprisingly, we could not confirm association of IGF2BP1 with the CTNNB1-encoding mRNA providing further evidence that the protein does not regulate CTNNB1 expression in HEK293 cells (Figure 2E).

The mRNA decay as well as RIP studies indicated that IGF2BP1 interfered with LEF1 mRNA degradation by associating with LEF1-encoding transcripts. Although IGF2BP1 was suggested to prevent MYC or PTEN mRNA degradation by associating with the respective coding regions of these transcripts, the protein was proposed to prevent decay of the CD44 mRNA via the 3′-UTR. In contrast to the MYC or PTEN mRNAs, no significant enrichment of rare codons was observed in the coding region of LEF1 mRNAs (data not shown). However, recent PAR-CLIP studies identified various putative association sites for IGF2BPs in the 3′-UTR of LEF1 encoding mRNAs, which with the exception of a small 5′-region is shared by all reported LEF1 transcripts (42). This suggested that IGF2BP1 controlled the fate of LEF1 mRNAs essentially via the 3′-UTR. To test this in further detail, the activity of luciferase reporters harboring either of the two so far reported LEF1-3′UTRs was analyzed on IGF2BP1 knockdown. The activity of the reporter comprising the BGH-derived 3′-UTR was only modestly decreased by IGF2BP1 depletion. On the contrary, the activity of the two analyzed LEF1 reporters was significantly reduced by IGF2BP1 depletion (Figure 2F). Reporter activity remained largely unaffected by the knockdown of IGF2BP2 or IGF2BP3, as observed for steady levels of LEF1 mRNAs (Supplementary Figure S2C).

**Figure 3.**
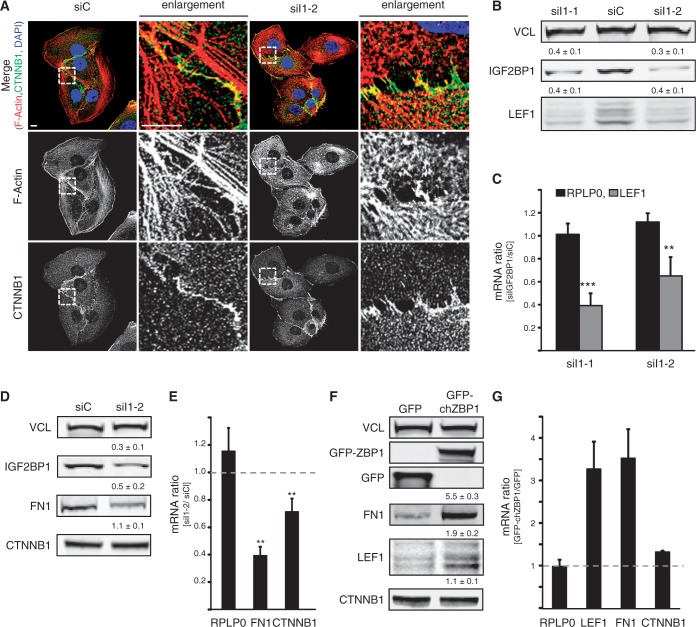
IGF2BP1 promotes the expression of LEF1 and FN1 in U2OS cells. (**A**) U2OS cells were transfected with IGF2BP1-directed (siI1-2) or control (siC) siRNAs for 72 h. Cell morphology and the actin cytoskeleton were analyzed by monitoring CTNNB1 localization using immunostaining or phalloidin labeling, respectively. Nuclei were labeled by DAPI. Enlargements of boxed regions (left panel) are shown in the right panels (enlargement). Bars, 10 µm. (**B** and **C**) The abundance of LEF1 protein and mRNA in response to IGF2BP1 knockdown (siI1-1 and siI1-2) was analyzed 72 h post-transfection by western blotting (B) or qRT-PCR (C), respectively. VCL served as the loading control to determine protein abundance relative to controls (siC), as is indicated above panels (B). LEF1 mRNA levels were determined relative to siC-transfected controls by the ΔΔC_t_-method using PPIA for normalization. RPLP0 mRNA served as a control. (**D** and **E**) FN1 and CTNNB1 protein (D) and mRNA (E) abundance was analyzed in U2OS cells 72 h post-transfection of indicated siRNAs as described in (B and C). CDH1 was not detectable in U2OS cells. (**F** and **G**) FN1, CTNNB1 and LEF1 protein (F) and mRNA (G) levels were investigated in U2OS cells stably transfected with GFP-ZBP1, the chicken ortholog of IGF2BP1 or GFP. Protein and mRNA abundance was essentially analyzed as described in (B and C). Statistical significance was determined by Student’s *t*-test: **P < 0.005; ***P < 0.0005. Error bars indicate SD of at least three independent analyses.

To exclude that IGF2BP1-facilitated regulation of LEF1 expression was exclusively observed in HEK293 cells, we analyzed how IGF2BP1 modulates mesenchymal properties and LEF1 synthesis in U2OS cells. Notably, we recently demonstrated that IGF2BP1 promotes U2OS migration and cell-matrix contact formation, two *bona fide* mesenchymal cell properties (23,25). In contrast to HEK293 cells, the depletion of IGF2BP1 had an only marginal effect on the morphology of U2OS cells, although actin fiber integrity was severely compromised as previously reported (Figure 3A). Despite only modest morphological alterations, IGF2BP1 depletion caused a decrease in LEF1 as well as FN1 mRNA and protein levels, whereas CTNNB1 protein abundance remained largely unaffected in U2OS cells (Figure 3B–E). Owing to its low abundance, altered expression of CDH1 could not be evaluated in U2OS cells (data not shown). To validate that IGF2BP1 promotes the expression of LEF1 and FN1, the chicken ortholog of human IGF2BP1, termed ZBP1, was stably expressed in U2OS cells, which compared with HEK293 cells express significantly lower levels of IGF2BP1 (8). In comparison with U2OS cells stably expressing GFP, GFP-tagged ZBP1 enhanced the expression of both, LEF1 and FN1 (Figure 3F and G). The expression of RPLP0, VCL as well as CTNNB1 remained essentially unaffected on the protein as well as mRNA level. In summary, these studies revealed that IGF2BP1 enhances the expression of LEF1 and FN1 in HEK293 and U2OS cells, suggesting largely cell context independent regulatory mechanisms. IGF2BP1 interfered with LEF1 mRNA degradation in a 3′-UTR-dependent manner. In contrast, the protein indirectly enhanced FN1 expression, potentially by stimulating LEF1-dependent FN1 transcription.

### LEF1 promotes mesenchymal-like cell properties

**Figure 4.**
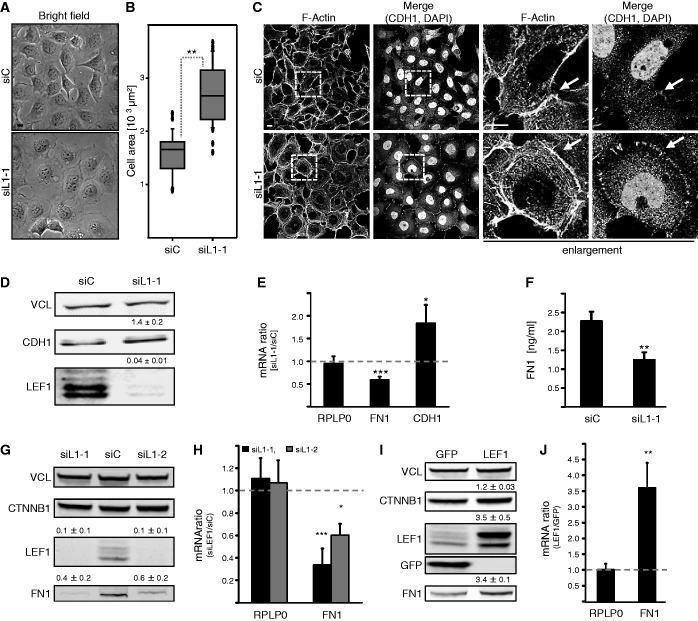
LEF1 promotes mesenchymal cell properties. HEK293A (**A–F**) or U2OS cells (**G** and **H**) were transfected with LEF1-directed (siL1-1 or siL1-2) or control (siC) siRNAs for 72 h. U2OS cells stably transfected with GFP or Flag-LEF1 were analyzed in (**I** and **J**). (A) Cell morphology was monitored by light microscopy; bar, 10 µm. (B) The size of adherent cells was determined by LSM microscopy, as described in Figure 1B. Please also refer to Supplementary Figure S2D. (C) The F-actin cytoskeleton and cell–cell contact formation was analyzed by phalloidin labeling and immunostaining for CDH1, where indicated nuclei were labeled by DAPI. Enlargements of boxed regions (left panels) are shown in right panels (enlargement); bars, 10 µm. The knockdown of LEF1 results in an enhanced recruitment of CDH1 to cell–cell contacts, whereas the F-actin cytoskeleton remains largely unaffected. (D) The abundance of CDH1 protein was analyzed by western blotting on LEF1 knockdown. Levels of LEF1 and CDH1 proteins were determined relative to controls (siC), as depicted above panels. VCL served as a loading control. (E) The abundance of FN1 and CDH1 mRNAs was investigated by qRT-PCR using the ΔΔC_t_-method and PPIA for normalization. RPLP0 served as a control. (F) Soluble FN1 concentrations in response to LEF1 knockdown were determined by ELISA as described in Figure 1G. (G and H) In U2OS cells transfected with indicated siRNAs, FN1 abundance was analyzed on protein (G) and mRNA (H) levels relative to controls as described in (D and E). (I and J) FN1, CTNNB1 and LEF1 protein (I) and FN1 mRNA (J) abundance was determined in U2OS cells stably transduced with Flag-LEF1 relative to GFP expressing controls. VCL served as loading control. Statistical significance was validated by Student’s *t*-test: *P < 0.05; **P < 0.005; ***P < 0.0005. Error bars indicate SD of at least three independent analyses.

Although IGF2BP1 could promote mesenchymal-like cell properties by enhancing the expression of LEF1 was investigated by analyzing the role of the transcriptional regulator LEF1 in HEK293 as well as U2OS cells. Similar to IGF2BP1 knockdown, the depletion of LEF1 resulted in significant morphological changes in HEK293 cells (Figure 4A). Cells appeared flattened, and the overall area covered by adherent cells was significantly increased, whereas the cell volume remained largely unaffected (Figure 4B and Supplementary Figure S2D and E). This suggested that the knockdown of LEF1 paralogues enhanced epithelial-like cell properties in HEK293 cells. In support of this, the localization of CTNNB1 and CDH1 to cell–cell contacts was obviously increased by LEF1 depletion (Figure 4C and Supplementary Figure S2G). Consistent with the assumption that LEF1 is not a potent repressor of CDH1 expression, CDH1 mRNA and protein levels were only modestly yet reproducibly upregulated by LEF1 knockdown (Figure 4D and E). More strikingly, LEF1 depletion resulted in a 2-fold reduction of FN1 mRNA and secreted protein levels (Figure 4E, F and Supplementary Figure S2F). As for IGF2BP1, this was further validated in U2OS cells. In these, FN1 mRNA and protein levels were decreased by LEF1 knockdown (Figure 4G, H). The opposite was observed on stable transfection of the longest LEF1 paralogue, which led to an upregulation of FN1 expression (Figure 4I and J). Hence, LEF1 promoted mesenchymal-like cell properties and enforced the expression of FN1 in HEK293 and U2OS cells.

### IGF2BP1 promotes FN1 and SNAI2 (SLUG) transcription through LEF1

IGF2BP1 promoted the expression of FN1 indirectly suggesting the protein induced FN1 transcription through LEF1, which was proposed to positively regulate FN1 mRNA synthesis (40,43). In accord, transient expression of the longest LEF1 paralogue enhanced FN1 mRNA abundance and secreted FN1 protein levels in HEK293 cells (Supplementary Figure S3A–C). Moreover, FN1 expression was restored in IGF2BP1 knockdown cells by the transient expression of LEF1 (Supplementary Figure S3D). Together, this supported the view that IGF2BP1 enhances the expression of FN1 through LEF1.

**Figure 5.**
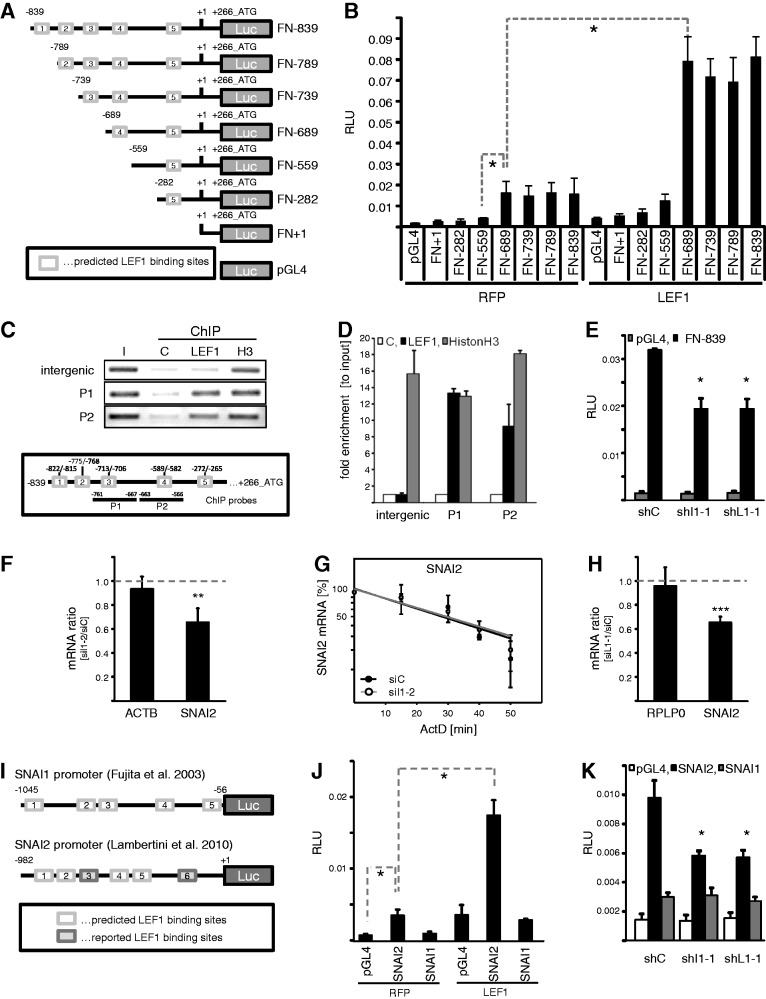
IGF2BP1 modulates FN1 and SNAI2 (SLUG) transcription via LEF1. (**A**) Schematic of luciferase reporters comprising the full-length *in silico* predicted (FN-839) or 5′-truncated fragments of the human FN1 promoter. The proposed transcription start is indicated by +1 with a reported 5′-UTR of 266 nt. Putative LEF1-binding sites predicted by ‘PROMO’ are depicted as white boxes with labels ‘1-5’ in 5′-to-3′ direction. (**B**) The Firefly luciferase activity of indicated promoter fragments or empty pGL4 vector was monitored in HEK293 cells on transient co-transfection with RFP or LEF1 for 30 h. Firefly activities were normalized by Renilla activities [relative luciferase units (RLU)], serving as internal controls. All reporters comprising the putative LEF1-binding site four showed promoter activity and were activated by LEF1. (**C** and **D**) Binding of endogenous LEF1 protein to the human FN1 promoter in HEK293 cells was assessed by ChIP. The association of endogenous LEF1 or histone H3 to the FN1 promoter was monitored by semi-quantitative (C) as well as quantitative PCR (D) using to FN1 promoter specific amplicons (P1 and P2, indicated in lower panel). An intergenic probe served as positive control. IgG-agarose was used to monitor unspecific binding (C, negative control). In (D), the enrichment of indicated genomic DNA fragments (P1 and P2) or the intergenic control (intergenic) was determined relative to the diluted input fraction (I) normalized by IgG-controls using the ΔC_t_-method. (**E**) HEK293 cells were co-transfected with FN-839 luciferase reporter and IGF2BP1-directed (shI1-1), LEF1-directed (shL1-1) or control shRNA encoding vectors for 48 h. RLUs were determined as described in (B). (**F**) HEK293 cells were transfected with IGF2BP1-directed (siI1-2) or control siRNAs (siC) for 72 h. The abundance of SNAI2 mRNA in response to IGF2BP1 knockdown was analyzed by qRT-PCR using the ΔΔC_t_-method and PPIA for normalization. ACTB served as control. (**G**) HEK293 cells transfected as in (F) were treated with ActD (5 µM) to block transcription for indicated times. SNAI2 mRNA turnover was analyzed by qRT-PCR using the ΔΔC_t_-method and PPIA for normalization. RNA decay is depicted in semi-logarithmic scale revealing no significant difference in mRNA turnover (*P*-value not shown). (**H**) HEK293 cells were transfected with LEF1-directed (siL1-1) or control siRNAs (siC) for 72 h. The abundance of SNAI2 mRNA in response to LEF1 depletion was analyzed by qRT-PCR using the ΔΔC_t_-method and PPIA for normalization. RPLP0 served as control. (**I**) Schematic of Firefly luciferase reporters comprising the SNAI1 or SNAI2 promoter sequences, as previously reported (37,45). Indicated putative LEF1-binding sites within the SNAI1 or SNAI2 promoter were predicted [white boxes; as described in (A)] or as previously reported [gray boxes, only for SNAI2; (37)]. (**J**) The Firefly activity of SNAI1 or SNAI2 promoter fragments cloned in pGL4 as well as the activity of empty pGL4 vector was monitored in HEK293 cells on transient co-transfection with RFP or LEF1 for 30 h. RLUs were determined as described in (B). LEF1 only enhanced the activity of the SNAI2 promoter. (**K**) HEK293 cells were co-transfected with SNAI1 or SNAI2 promoter reporters and indicated shRNA-encoding vectors for 48 h. RLUs were determined as described in (B). SNAI2 promoter activity was reduced by IGF2BP1 as well as LEF1 knockdown, whereas the SNAI1 reporter activity remained largely unaffected and was barely elevated compared with the empty control reporter. Statistical significance was validated by Student’s *t*-testing: *P < 0.05; ***P < 0.0005. Error bars indicate SD of at least three independent analyses.

*In silico* analyses of the genomic sequence upstream of the starting ATG of the human FN1 gene (Chr.2q34) suggested a putative minimal promoter of approximately 1 kb comprising five candidate LEF1-targeting sites (Figure 5A). To analyze whether LEF1 stimulates FN1 transcription through the FN1 promoter, the Renilla luciferase normalized activity of Firefly luciferase reporters driven by indicated fragments of the FN1 promoter were analyzed in HEK293 cells. In cells, co-transfected with red fluorescent protein (RFP), FN1 reporter activity was only observed for reporters comprising the predicted binding site four (Figure 5B, RFP). All shorter FN1 promoter fragments showed an only basal activity, which was indistinguishable from background activity determined for the control reporter lacking any promoter (Figure 5B, pGL4). Activity of reporters comprising binding site four was ∼4-fold increased by the transient expression of the longest LEF1 paralogue reported in human (Figure 5B, LEF1). Together, this suggested that LEF1 promotes FN1 transcription by associating with site four in the FN1 promoter. However, activity of the longest promoter reporter (FN-839) remained essentially unaffected by the deletion of site four, which could be due to the conditioning of binding by surrounding sequences (Supplementary Figure S3E). We therefore analyzed whether LEF1 associates with the FN1 promoter using ChIP studies (Figure 5C and D). Immunopurification of LEF1 followed by semi-quantitative PCR analyses revealed robust copurification of two genomic sequences located in the human FN1 promoter, whereas binding to intergenic elements was not observed (Figure 5C, P1-P2; schematic shows position of ChIP PCR amplicons and putative binding sites). Histone H3 served as a non-promoter selective positive and IgG-agarose as a negative control. Quantitative assessment of the ChIP analyses indicated selective binding of LEF1 to the FN1 promoter and suggested association at or in proximity to the predicted binding sites three and four (Figure 5D). Finally, IGF2BP1 as well as LEF1-dependent transcriptional regulation of FN1 was evaluated by determining the activity of the longest reporter (FN-839) in response to IGF2BP1 or LEF1 knockdown (Figure 5E). The depletion of both factors reduced the reporter activity significantly supporting the view that IGF2BP1 promoted FN1 transcription in a LEF1-dependent manner. However, these analyses could not exclude whether IGF2BP1 also modulates the expression of additional factors directly or indirectly regulating the transcription of FN1.

Recent studies revealed that LEF1 enhances the expression of two transcriptional ‘drivers’ of EMT [reviewed in (2)], ZEB2 and SNAI2 (SLUG), in breast carcinoma-derived MDA-MB-231 cells (44). Moreover, it was postulated that LEF1 enhances SNAI2 transcription (37). Accordingly, it was tempting to speculate that IGF2BP1, by enhancing the expression of LEF1, also induces the expression of other pro-mesenchymal transcriptional regulators. To evaluate this hypothesis, SNAI2 expression in HEK293 cells was first determined in response to IGF2BP1 depletion (Figure 5F and Supplementary Figure S3F). Steady state SNAI2 protein as well as mRNA levels were reduced on IGF2BP1 knockdown. However, SNAI2 mRNA turnover remained unchanged, as determined by mRNA decay analyses in response to blocking transcription by ActD (Figure 5G). Moreover, direct association of IGF2BP1 was only observed for the LEF1, but not the SNAI2 mRNA by RIP analyses (Supplementary Figure S3H). This suggested that IGF2BP1 sustains SNAI2 expression indirectly, potentially by promoting the expression of LEF1. To test this directly, SNAI2 expression was monitored on LEF1 knockdown in HEK293 cells (Figure 5H and Supplementary Figure S3G). As expected and previously reported for breast cancer-derived tumor cells, LEF1 depletion resulted in decreased steady state SNAI2 protein as well as mRNA levels. Whether LEF1 similar to FN1 controls SNAI2 expression by promoting the transcription of this ‘EMT-driver’ was analyzed via a previously validated luciferase reporter comprising the SNAI2-promoter [Figure 5I, (37)]. A reporter driven by the promoter of SNAI1, another ‘EMT-driving’ transcriptional regulator, served as control [Figure 5I, (45)]. In accord with previous studies, transiently expressed LEF1 enhanced the activity of the SNAI2, but not the SNAI1 reporter (Figure 5J). The opposite was observed on IGF2BP1 as well as LEF1 knockdown. Although SNAI2 reporter activity was decreased by the depletion of IGF2BP1 or LEF1, SNAI1 reporter activity remained unaffected (Figure 5K). Notably, we also attempted to validate direct association of LEF1 with the SNAI2 promoter by ChIP, as previously demonstrated in human osteoblasts (46). However, we could not confirm association of LEF1 with the SNAI2 (46) or CDH1 (43) promoter in HEK293 cells (Supplementary Figure S3I). Recent studies reported that the expression of ‘EMT-driving’ transcriptional regulators like ZEBs, SNAILs and potentially LEF1 is essentially modulated by regulatory feedback loops facilitated via microRNAs [reviewed in (3,4)]. To evaluate whether LEF1 could regulate the expression of SNAI2 in a microRNA-dependent manner, the activity of reporters comprising the SNAI2 3′-UTR was analyzed in response to LEF1 overexpression (Supplementary Figure S3J). Activity of the SNAI2 3′-UTR comprising reporter was significantly reduced compared with the control reporter supporting inhibition of SNAI2 expression by microRNAs, for instance the miR-34 family (5). The overexpression of LEF1, however, had no significant effect on reporter activity. This suggested that LEF1 promotes SNAI2 transcription rather than modulating the post-transcriptional fate of the SNAI2 mRNA in HEK293 cells. Whether this regulation is facilitated via direct association with the SNAI2 promoter or in an indirect manner via additional yet to identify factors remained elusive, as no association of LEF1 with the SNAI2 promoter could be determined by ChIP. Taken together, these analyses indicated that IGF2BP1 promotes the expression of two ‘EMT-driving’ transcriptional regulators, LEF1 and SNAI2. IGF2BP1 enhanced LEF1 expression by interfering with LEF1 mRNA degradation resulting in presumably LEF1-dependent enhancement of FN1 transcription and SNAI2 expression.

### IGF2BP1 sustains mesenchymal-like tumor cell properties

**Figure 6.**
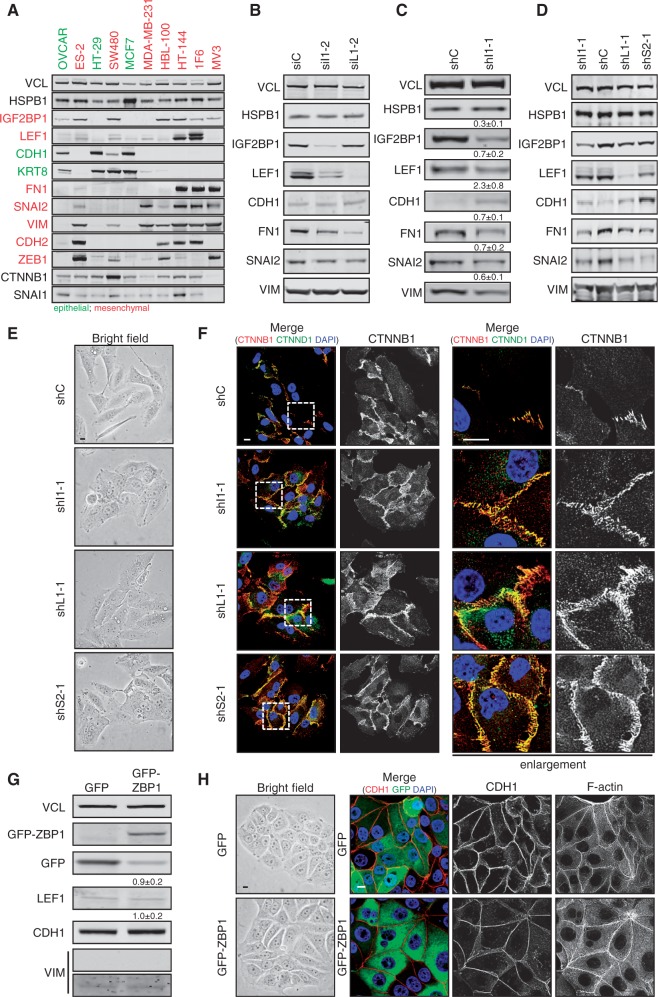
IGF2BP1 sustains mesenchymal-like tumor cell properties. (**A**) Indicated tumor-derived cell lines were cultured (48 h) and harvested at 80% confluence before analyzing the abundance of indicated proteins by western blotting. Epithelial-like cell lines and marker proteins are labeled in green. Mesenchymal-like cell lines and marker proteins are depicted in red. VCL and HSPB1 (HSP27) served as loading controls. IGF2BP1 is almost exclusively expressed in the following tumor-derived mesenchymal-like cells: ES-2 ovarian carcinoma (ATCC#: CRL-1978); SW480 colorectal carcinoma (ATCC#: CCL228); MDA-MB-231 (ATCC#: HTB-26) and HBL-100 (ATCC#: HTB-124) mammary carcinoma; 1F6 (no ATCC# available) and HT-144 (ATCC#: HTB-63) melanoma. In epithelial-like adenocarcinoma-derived cells [OVCAR (ATCC#: HTB-161) ovarian adenocarcinoma; MCF7 (ATCC#: HTB-22) breast adenocarcinoma; HT-29 (ATCC#: HTB-38) colorectal adenocarcinoma], expression of IGF2BP1 was only observed in OVCAR cells. (**B**) Melanoma-derived HT-144 cells were transiently transfected with indicated siRNAs for 72 h. The abundance of indicated proteins was analyzed by western blotting. IGF2BP1 as well as LEF1 depletion result in reduced FN1 and SNAI2 protein abundance, whereas CDH1 and VIM levels remain essentially unchanged. (**C–F**) HT-144 cells were stably transduced by lentiviral vectors encoding IGF2BP1 (shI1-1), LEF1 (shL1-1), SNAI2 (shS2-1) directed or control (shC) shRNAs. Three weeks after transduction, cells were cultured for 48 h before analyzing protein abundance by western blotting with indicated antibodies (C and D). Protein abundance on IGF2BP1 knockdown was determined relative to controls (siC) using VCL and HSPB1 for cross-normalization, as indicated above panels (C). Standard deviation of at least three independent analyses is shown. The stable knockdown of SNAI2, LEF1 and IGF2BP1 promotes the expression of the epithelial marker CDH1, whereas all mesenchymal marker proteins were reduced. Cell morphology was monitored by bright field microscopy (E). Cells were cultured on collagen coated coverslips for 48 h before immunostaining of CTNNB1 and CTNND1 (p120 Catenin) to label cell–cell contacts (F). Enlargements of boxed regions (left panels) are shown in right panels (enlargement). All three stable knockdowns promote the formation of cell–cell contacts, suggesting an enhancement of epithelial-like cell morphology. (**G** and **H**) MCF7 cells were stably transduced with GFP-ZBP1 (the chicken ortholog of IGF2BP1) or GFP. Three weeks after transduction, cells were cultured for 48 h before determining the abundance of indicated proteins by western blotting (G). Cell morphology was monitored by bright field microscopy (H, left panel) and immunostaining for CDH1 as well as labeling of F-actin by phalloidin (H, right panel). Neither CDH1 expression nor cell-cell contact formation is compromised by GFP-ZBP1, although cell size appeared modestly increased. Bars, 10 µm.

IGF2BP1 promotes tumor cell migration [reviewed in (23)], sustained the expression of pro-mesenchymal transcriptional regulators, and its depletion interfered with mesenchymal-like cell morphology in HEK293 as well as U2OS cells. This supported the view that IGF2BP1 serves an essential pro-mesenchymal role in tumor-derived cells, suggesting the protein as a mesenchymal marker. To evaluate this assumption, we analyzed the expression of IGF2BP1 and mesenchymal as well as epithelial markers in a panel of 10 cell lines derived from distinct tumors or metastases (Figure 6A). Except ovarian carcinoma-derived OVCAR cells, which expressed CDH1 and KRT8 but barely any of the analyzed mesenchymal markers, significant IGF2BP1 expression was only observed in mesenchymal-like tumor- or metastases-derived cells. With the exception of breast carcinoma-derived MDA-MB-231 cells, IGF2BP1 expression was well correlated with the expression of the mesenchymal marker vimentin (VIM). This provided further evidence for an essential function of IGF2BP1 in inducing and/or sustaining mesenchymal-like properties in various, although not all, mesenchymal-like tumor cells. To test this in further detail, the role of IGF2BP1 was analyzed in melanoma-derived HT-144 and 1F6 cells as well as ovarian carcinoma-derived ES-2 cells. Notably, we recently demonstrated that IGF2BP1 promotes ES-2 cell migration (25). As observed in HEK293 or U2OS cells, the transient knockdown of IGF2BP1 as well as LEF1 led to reduced FN1 as well as SNAI2 expression in all three cell lines. VIM or CDH1 protein abundance remained largely unaffected (Figure 6B and Supplementary Figure S4A and D). Despite unaffected expression of these markers, cell morphology was significantly altered, and cell–cell contact formation, as determined by CTNNB1 localization at cell borders, appeared increased on the depletion of IGF2BP1 or LEF1 (Supplementary Figures S4B, C, E, F and S5A and B). Whether the stable knockdown of IGF2BP1, LEF1 or SNAI2 promoted epithelial-like cell characteristics and marker expression in a more ‘sustained’ manner was analyzed in HT-144 cells transduced with shRNA-encoding lentiviral vectors. The stable knockdown of IGF2BP1 led to ∼3-fold reduced IGF2BP1 abundance, which was associated with a significant downregulation of LEF1, FN1, SNAI2 and also VIM. Expression of the epithelial marker CDH1 was modestly, but reproducibly, increased (Figure 6C and D). The same was observed on the stable knockdown of LEF1 and SNAI2, although significant upregulation of CDH1 was only observed on SNAI2 depletion confirming the pivotal role of this transcriptional ‘EMT-driver’ in the repression of CDH1. Notably, the knockdown of LEF1 as well as SNAI2 also interfered with IGF2BP1 expression, which could indicate that IGF2BP1 expression is controlled by ‘EMT-driving’ transcriptional regulators like LEF1 and/or SNAI2. Consistent with the observed shift in the expression of mesenchymal versus epithelial markers, the knockdown of all three factors caused severe morphological changes in HT-144 with an increase of CTNNB1 positive cell–cell contact sites (Figure 6E and F). These findings indicated that IGF2BP1 sustains the expression of mesenchymal markers and mesenchymal-like cell morphology involving the enhancement of LEF1 and SNAI2 expression. However, it remained elusive whether IGF2BP1 also induces mesenchymal-like cell properties or even EMT. To address this in further detail, we transduced breast cancer-derived MCF7 cells, which express IGF2BP1 at barely detectable levels (see Figure 6A), with lentiviral vectors encoding either GFP or GFP-fused ZBP1, the chicken ortholog of IGF2BP1. Two to three weeks after infection, the expression of low abundant LEF1 as well as highly expressed CDH1 remained essentially unaffected by GFP-ZBP1 (Figure 6G). Likewise, no significant increase was observed for the expression of VIM, which was barely detectable to begin with. In accord with the unaltered expression of mesenchymal and epithelial markers, the overall cell morphology remained essentially unchanged with no obvious defect in cell–cell contact formation, as evidenced by CDH1 immunostaining (Figure 6H). This was further analyzed in epithelial MDCK, which, despite their epithelial morphology, expresses IGF2BP1 (Supplementary Figure S7D). The stable expression of GFP-ZBP1 using lentiviral transduction significantly increased the size of adherent MDCK cells (Supplementary Figure S7A and B). However, cell–cell contact formation and the expression of the epithelial marker CDH1 or the mesenchymal marker VIM remained unaffected by ZBP1 (Supplementary Figure S7C and D). Finally, we analyzed whether the forced expression of ZBP1 increased the migratory potential of MDCK cells. Wound closure studies revealed that the motility of MDCK cells was unchanged (Supplementary Figure S7E and F). Taken together, this indicated that IGF2BP1 rather sustains than induces mesenchymal-like cell properties in tumor-derived or immortalized cells. However, its potential role in inducing mesenchymal-like cell properties or even EMT has been validated here for only two cell lines (MCF7 and MDCK). In contrast, IGF2BP1-dependent sustainment of mesenchymal cell properties could be validated for all so far analyzed mesenchymal-like tumor-derived cells expressing IGF2BP1.

### IGF2BP1 promotes migration and mesenchymal cell morphology through LEF1 and SNAI2

**Figure 7.**
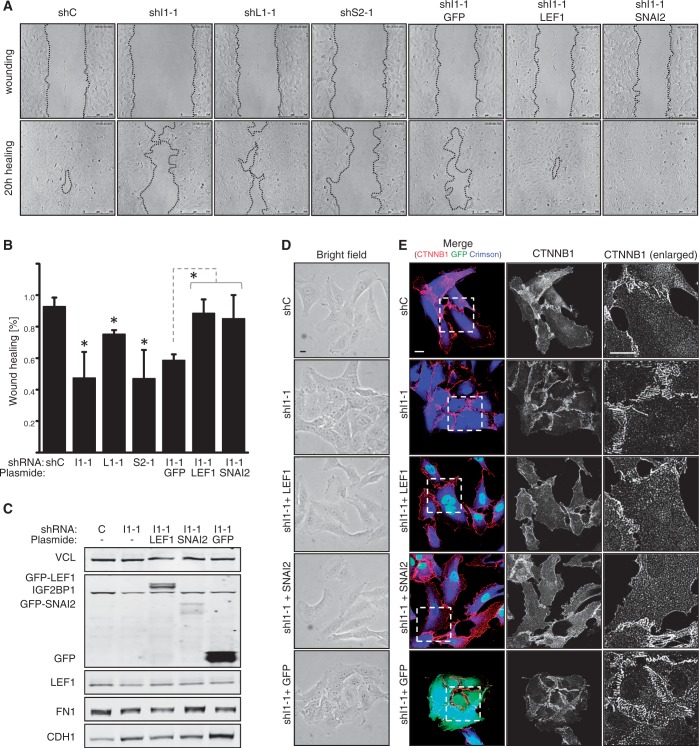
IGF2BP1 promotes migration and mesenchymal-like cell morphology via LEF1 and SNAI2. HT-144 cells were stably transduced by lentiviral vectors encoding IGF2BP1 (shI1-1), LEF1 (shL1-1), SNAI2 (shS2-1) directed or control (shC) shRNAs, where indicated IGF2BP1 knockdown populations were transduced with GFP, GFP-LEF1 or GFP-SNAI2 cDNA-encoding lentiviral vectors 3 weeks after the infection with shRNA-encoding vectors. (**A** and **B**) Cell migration was analyzed using wound closure analyses monitored by time lapse microscopy over 20 h (A; Bars, 250 µm). Cell migration was assessed by quantitative means relative to cells transduced with control shRNA (shC) using automated segmentation algorithms (B), as recently described (39). SD was determined over three independent analyses. Statistical significance was validated by Student’s *t*-test: *P < 0.05. The depletion of IGF2BP1, LEF1 or SNAI2 significantly reduces cell migration. Migration of IGF2BP1 knockdown populations is restored by the expression of LEF1 or SNAI2. (**C**) The abundance of indicated epithelial or mesenchymal markers was analyzed by western blotting in indicated cell populations as described in Figure 6C. (**D** and **E**) Cell morphology of indicated cell populations was monitored by bright field microscopy (D) and immunostaining for CTNNB1 (E). Transduction with shRNA-encoding lentiviral vectors was monitored by E2-Crimson (pseudo-colored in blue), whereas the expression of GFP-LEF1, GFP-SNAI2 or GFP is depicted in green. Mesenchymal-like cell morphology was essentially restored by the stable expression of GFP-LEF1 or GFP-SNAI2 in IGF2BP1 knockdown populations. Enlargements of boxed regions are shown in the right panels (E, enlarged). Bars, 10 µm.

In previous studies, we demonstrated that IGF2BP1 promotes the migratory potential as well as cell-matrix contact formation of various tumor-derived cells. These analyses indicated that IGF2BP1 modulates these *bona fide* mesenchymal-like cell properties by fine tuning actin dynamics in a MK-signaling dependent manner. However, pharmacological inhibition of MK-signaling only partially, although significantly, restored cell migration and the formation of focal adhesions (23,25). Therefore, it was tempting to speculate that the role of IGF2BP1 in directing cell migration and sustaining mesenchymal-like cell properties is also modulated through the pro-mesenchymal regulators LEF1 and/or SNAI2. Accordingly, cell migration was analyzed by wound healing analyses in HT-144 cells using automated segmentation algorithms to quantify wound closure (39). Consistent with recent findings, stable IGF2BP1 knockdown reduced wound closure by ∼2-fold (Figure 7A and B). Significantly reduced cell migration was also observed on the stable knockdown of LEF1 or SNAI2, although cell migration was only moderately affected by LEF1 depletion when compared with the knockdown of IGF2BP1 or SNAI2. Whether LEF1 or SNAI2 could recover IGF2BP1 knockdown-induced impairment of tumor cell migration was determined by their stable expression in IGF2BP1-depleted cells. In comparison with IGF2BP1 knockdown cells stably expressing GFP, cell migration was restored substantially by the forced expression of either LEF1 or SNAI2. Surprisingly, however, the stable expression of LEF1 or SNAI2 had only moderate effects on FN1 or CDH1 abundance in IGF2BP1 knockdown cells (Figure 7C). Despite largely unaltered expression of these markers, mesenchymal-like cell morphology with reduced cell–cell contacts was observed on the expression of LEF1 and SNAI2 in cells stably transduced with IGF2BP1-directed shRNAs (Figure 7D and E). IGF2BP1 knockdown cells showed pronounced cell–cell contact formation with increased recruitment of CTNNB1 to cell–cell contact sites. This was correlated with an enhanced association of cells observed by bright field analyses (Figure 7D). The stable expression of LEF1 or SNAI2 induced a more mesenchymal-like appearance of cell morphology with reduced association of cells and less striking recruitment of CTNNB1 to cell borders. CTNNB1 appeared to be enriched in the cytoplasm and was even observed, although at moderate levels, in the nucleus of some cells transduced with LEF1 or SNAI2 (Figure 7E). Hence, although FN1 and CDH1 are presumably not the key markers involved, these findings supported the view that the role of IGF2BP1 in promoting tumor cell migration and sustaining mesenchymal-like cell morphology involves the upregulation of LEF1 and SNAI2.

## DISCUSSION

This study identifies a novel mechanism by which the RBP IGF2BP1 sustains mesenchymal tumor cell properties and promotes the migration of tumor-derived cells *in vitro*. This regulation is essentially facilitated by IGF2BP1-directed upregulation of the ‘EMT-driving’ transcriptional regulators LEF1 and SNAI2 [reviewed in (1,2)]. IGF2BP1 interferes with LEF1 mRNA degradation in a 3′-UTR dependent manner resulting in the enhancement of LEF1 expression. This in turn promotes transcription of the extracellular matrix component FN1, a *bona fide* mesenchymal marker. Moreover, IGF2BP1 indirectly promotes SNAI2 (SLUG) transcription, presumably in a LEF1-dependent manner. In agreement with this pro-mesenchymal role of IGF2BP1, the protein is predominantly observed in mesenchymal-like tumor-derived cells in which it enhances motility. In addition to controlling actin dynamics (23,25), IGF2BP1 sustains mesenchymal cell properties and modulates tumor cell migration also through the upregulation of LEF1 and SNAI2. However, the stable expression of IGF2BP1 in epithelial-like MCF7 tumor-derived cells or kidney-derived MDCK cells failed to induce EMT or a significant upregulation of mesenchymal marker expression. This indicates that IGF2BP1 sustains but does not induce pro-mesenchymal gene expression by promoting the expression of ‘EMT-driving’ transcriptional regulators at the post-transcriptional level. This results in elevated tumor cell migration and potentially enhances the invasive potential of tumor cells (Supplementary Figure S8).

*In vitro*, IGF2BP1 was identified as a pro-migratory RBP, which in various tumor-derived cells promotes both the velocity and directionality of migration. Expression profiling of primary tumors and metastases provided substantial evidence for an upregulation or even *de novo* synthesis of IGF2BP1 and/or IGF2BP3 in almost all solid cancers analyzed so far [reviewed in (8,23)]. Together, this suggests a fundamental role of IGF2BP1 and potentially IGF2BP3 in tumor cell dissemination, which presumably is observed in a broad variety of tumors. In support of this, it was reported that the transgenic expression of mouse IGF2BP1 (CRD-BP) in mammary tissue promotes the formation of primary breast carcinomas as well as metastases (47). In contrast, IGF2BP1 was proposed to interfere with the *in vitro* migration of breast cancer-derived cells and enhance the formation of cell–cell contacts (22,36). Hence, it remained contradictory whether IGF2BP1 promotes mesenchymal-like properties of tumor cells and modulates their migration in a largely cell context independent manner. Therefore, we addressed how perturbing IGF2BP1 expression in tumor-derived cells and non-tumorigenic HEK293 cells, which express exceedingly high levels of IGF2BP1, affects mesenchymal- versus epithelial-like cell properties. In HEK293 cells, depletion of the protein induced severe morphological changes, which were associated with an increase in cell–cell contact formation, moderate upregulation of the epithelial marker CDH1 and significant downregulation of the mesenchymal marker FN1. These pro-epithelial changes in cell morphology and marker expression were observed to varying extend for all analyzed tumor-derived cells expressing IGF2BP1. Accordingly, we propose that IGF2BP1 is a pro-mesenchymal marker that sustains mesenchymal-like cell properties and promotes migration of tumor-derived cells in a largely context-independent manner.

IGF2BPs control the cytoplasmic fate of specific target mRNAs by regulating their turnover, translation and/or transport [reviewed in (8,10)]. This implied that the pro-mesenchymal role of IGF2BP1 is facilitated by regulating the fate of target transcripts encoding either regulators or markers of pro-mesenchymal gene expression signatures. In recent studies, we identified various novel candidate target mRNAs of IGF2BP1 by using a loss-of-function screen in stressed U2OS cells (25). These analyses suggested mRNAs encoding the pro-mesenchymal or ‘EMT-driving’ transcriptional regulator LEF1 as candidate target transcripts of IGF2BP1. Analyses of how IGF2BP1 modulates LEF1 mRNA fate revealed that the protein interferes with the degradation of LEF1 mRNAs resulting in elevated expression of this TCF family member. Similar to the IGF2BP-directed control of CD44 expression (28), IGF2BP1 prevents degradation of LEF1 mRNAs via the 3′-UTR essentially shared by all four reported human LEF1 transcripts. Notably, IGF2BP1-controlled LEF1 expression could be validated by loss- as well as gain-of-function analyses and was observed in all mesenchymal-like tumor-derived cells analyzed in this study. This suggests LEF1 as a prime, although not exclusive candidate, through which IGF2BP1 promotes mesenchymal-like tumor cell properties.

The TCF/LEF family of transcriptional regulators was identified as a key mediator of both Wnt/CTNNB1- or TGFB/SMAD-dependent developmental and malignant EMT [reviewed in (2,48)]. In agreement with ‘EMT-driving’ functions, LEF1 depletion was associated with reduced FN1 expression in all cells analyzed in this study, whereas the opposite was observed by stable LEF1 expression in U2OS or HEK293 cells. This supports LEF1-dependent upregulation of FN1 transcription observed by promoter-reporter and ChIP studies. However, LEF1 is presumably not the only mediator of FN1 expression, as FN1 is also expressed in cells lacking LEF1, for instance melanoma-derived MV3 cells. In contrast to FN1, LEF1 depletion or overexpression barely affected the expression of CDH1 supporting the view that LEF1 is not a potent repressor of CDH1 transcription. This is surprising, as LEF1 was shown to enhance the transcription of SNAI2 (SLUG) a *bona fide* ‘EMT-driving’ regulator suppressing CDH1 expression (37,49). However, LEF1-driven upregulation of SNAI2 may simply be too moderate to push SNAI2 abundance to levels sufficient for CDH1 repression. Consistent with IGF2BP1-promoted expression of LEF1, IGF2BP1 and LEF1 depletion resulted in reduced SNAI2 expression, presumably owing to reduced transcription. Direct regulation of SNAI2 mRNA fate by IGF2BP1 could be excluded, as the protein neither associates with the SNAI2 mRNA nor modulates its turnover. Thus, taken together, our studies suggest that IGF2BP1 can promote the expression of mesenchymal markers and modestly interfere with the expression of epithelial markers. This regulation is likely to be facilitated via LEF1 and SNAI2 but presumably also involves additional regulators like ZEBs or TWISTs. Notably, we have substantial evidence that IGF2BP1 promotes the expression of ZEB1, another potent ‘EMT-driving’ transcriptional regulator, in anaplastic thyroid carcinoma-derived tumor cells (Mensch *et al.*, in preparation). Importantly, in none of the analyzed tumor-derived cells, IGF2BP1 depletion was sufficient to induce upregulation of epithelial markers to a level expected for MET. Likewise, stable IGF2BP1 or ZBP1 expression in epithelial-like MCF7 or MDCK cells failed to induce EMT. This supports the view that IGF2BP1 sustains mesenchymal-like cell properties and potentially EMT-induced reprograming of gene expression at the post-transcriptional level. However, it fails to induce this reprograming, as this requires the induction of powerful upstream drivers at the transcriptional and/or epigenetic level. Along these lines, even the stable expression of *bona fide* ‘EMT-driving’ transcriptional regulators like SNAI2 failed to induce EMT in MCF7 cells, although CDH1 levels were significantly reduced, and cell size was markedly increased (Supplementary Figure S6A–C). This is consistent with the assumption that in some or even most tumor-derived cells, one ‘EMT-driver’ is insufficient to induce trans-differentiation. Moreover, this provides further support for the view that RBPs, which only fine tune gene expression at the post-transcriptional level, with few exceptions like SXL in *Drosophila*, simply lack the potency to induce a complete reprograming of gene expression signatures. This of course does not contradict a significant influence in the sustainment of altered gene expression at the post-transcriptional level.

Strikingly, we observed that IGF2BP1-facilitated modulation of tumor cell migration involves IGF2BP1-directed control of LEF1 and SNAI2 expression. This conclusion is supported by the finding that reduced migration of HT-144 cells on IGF2BP1 knockdown was completely restored by the stable expression of LEF1 or SNAI2. Notably, this is intriguingly consistent with the reported role of both factors in tumor cell invasion and metastasis, which essentially, although by far not exclusively, relies on the migratory capability of tumor cells [e.g. (50,51)]. In support of the interdependence of EMT, enhanced migratory potential and metastasis, the stable expression of LEF1 or SNAI2 also pronounced more mesenchymal-like cell morphology with reduced cell–cell contact formation on stable IGF2BP1 knockdown in HT-144 cells. Hence, although IGF2BP1 failed to induce EMT and sustained the expression of mesenchymal markers only moderately, the post-transcriptional fine tuning of gene expression facilitated by IGF2BP1 is sufficient to substantially impact cell morphology and tumor cell migration. This suggests that IGF2BP1 modulates various pro-mesenchymal regulatory networks including the control of actin dynamics (23,25) and the sustainment of pro-mesenchymal gene expression signatures driven by transcriptional regulators like LEF1 or SNAI2. In view of the proposed multitude of target mRNAs regulated by IGF2BP1 and IGF2BP3 (42), these findings suggest a fundamental role of both factors in promoting tumor cell aggressiveness and invasive potential in a largely tumor origin-independent manner. Future studies will now have to reveal whether this conclusion can be validated *in vivo* by testing to which extent both proteins promote metastasis and via which target mRNAs or signaling networks this regulation is facilitated. We expect that such analyses will indicate IGF2BP1 and IGF2BP3 as useful biomarkers for evaluating tumor aggressiveness and will reveal avenues to pursue analyzing their suitability for targeted therapy. The latter would benefit substantially from the fact that both factors are essentially *de novo* synthesized in various tumors, whereas they are barely expressed in the vast majority of adult tissues (8).

## SUPPLEMENTARY DATA

Supplementary Data are available at NAR Online: Supplementary Tables 1–4 and Supplementary Figures 1–9.

## FUNDING

Funding for open access charge: Deutsche Forschungs Gemeinschaft (DFG) [HU1547/3-1, HU1547/2-2 and GRK1591 to S.H.].

*Conflict of interest statement*. None declared.

## Supplementary Material

Supplementary Data
