# Supplementary material for: IGF2BP1 promotes mesenchymal cell properties and migration of tumor-derived cells by enhancing the expression of LEF1 and SNAI2 (SLUG)
Source: Nucleic Acids Res. 2013 May 15;41(13):6618–36. doi: 10.1093/nar/gkt410 (PMC3711427; doi:10.1093/nar/gkt410)
Supplement: Supplementary Data [file supp_41_13_6618__index.html]

IGF2BP1 promotes mesenchymal cell properties and migration of tumor-derived cells by enhancing the expression of LEF1 and SNAI2 (SLUG) — IGF2BP1 promotes mesenchymal cell properties and migration of tumor-derived cells by enhancing the expression of LEF1 and SNAI2 (SLUG) — Supplementary Data 

# IGF2BP1 promotes mesenchymal cell properties and migration of tumor-derived cells by enhancing the expression of LEF1 and SNAI2 (SLUG)

## Supplementary Data

files

**Files in this Data Supplement:**

- Supplementary Data - pdf file
- Supplementary Data - docx file
