# Supplementary material for: IGF2BP1 promotes mesenchymal cell properties and migration of tumor-derived cells by enhancing the expression of LEF1 and SNAI2 (SLUG)
Source: Nucleic Acids Res. 2013 May 15;41(13):6618–36. doi: 10.1093/nar/gkt410 (PMC3711427; doi:10.1093/nar/gkt410)
Supplement: Supplementary Data [file supp_gkt410_nar-02409-v-2012-File003.pdf]

A

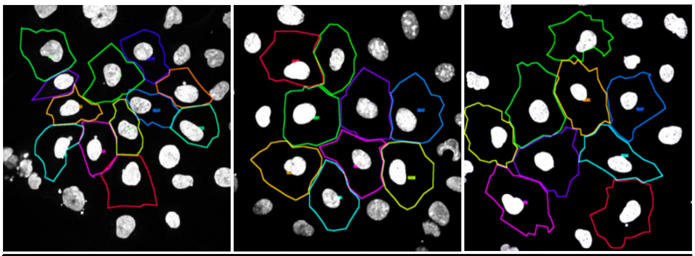

		N	Cell area [µm²]	Std Dev
1	siC	31	1573.706	585.51
2	siI1-2	32	2542.174	667.23
3	siI1-3	16	2854.387	338.69

B

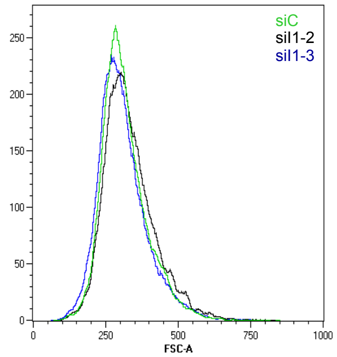

C

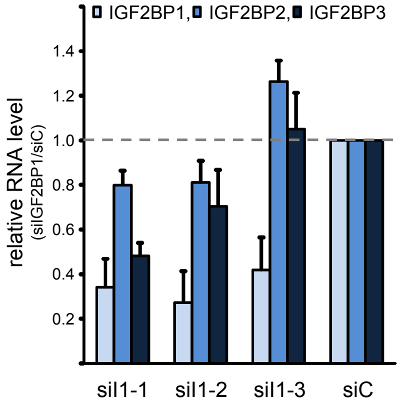

D

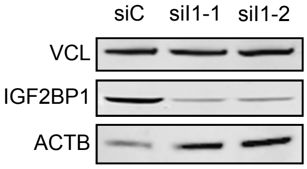

E

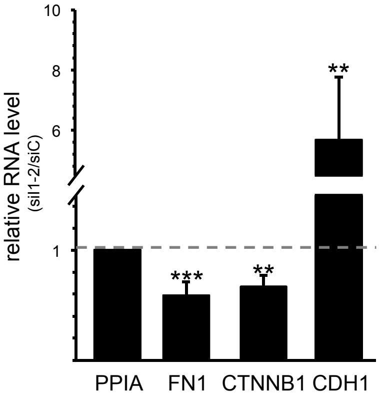

F

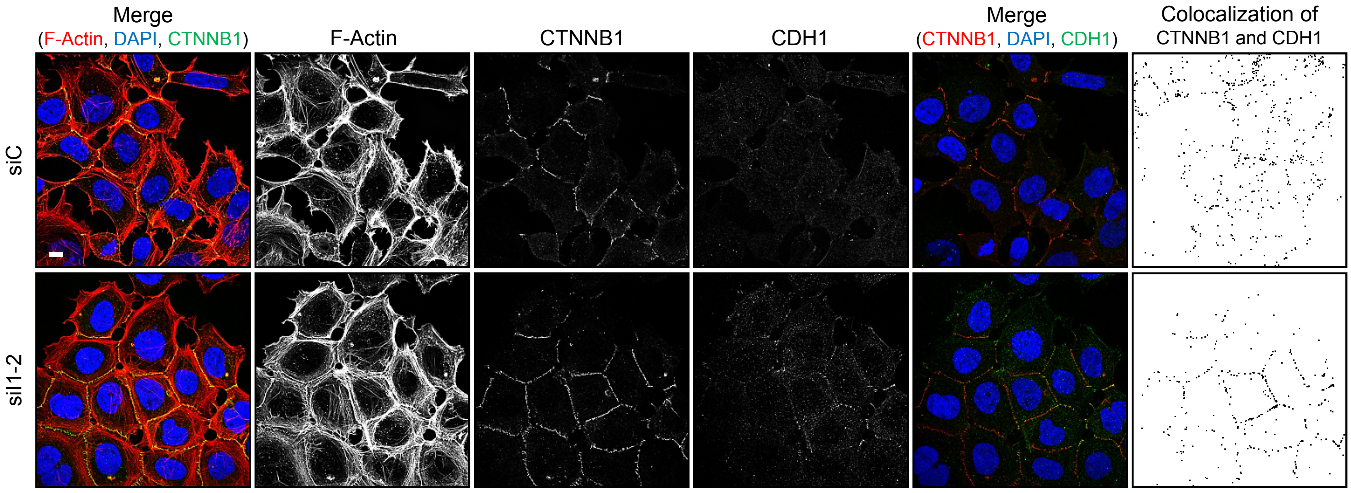

Figure S1, Zirkel et al.

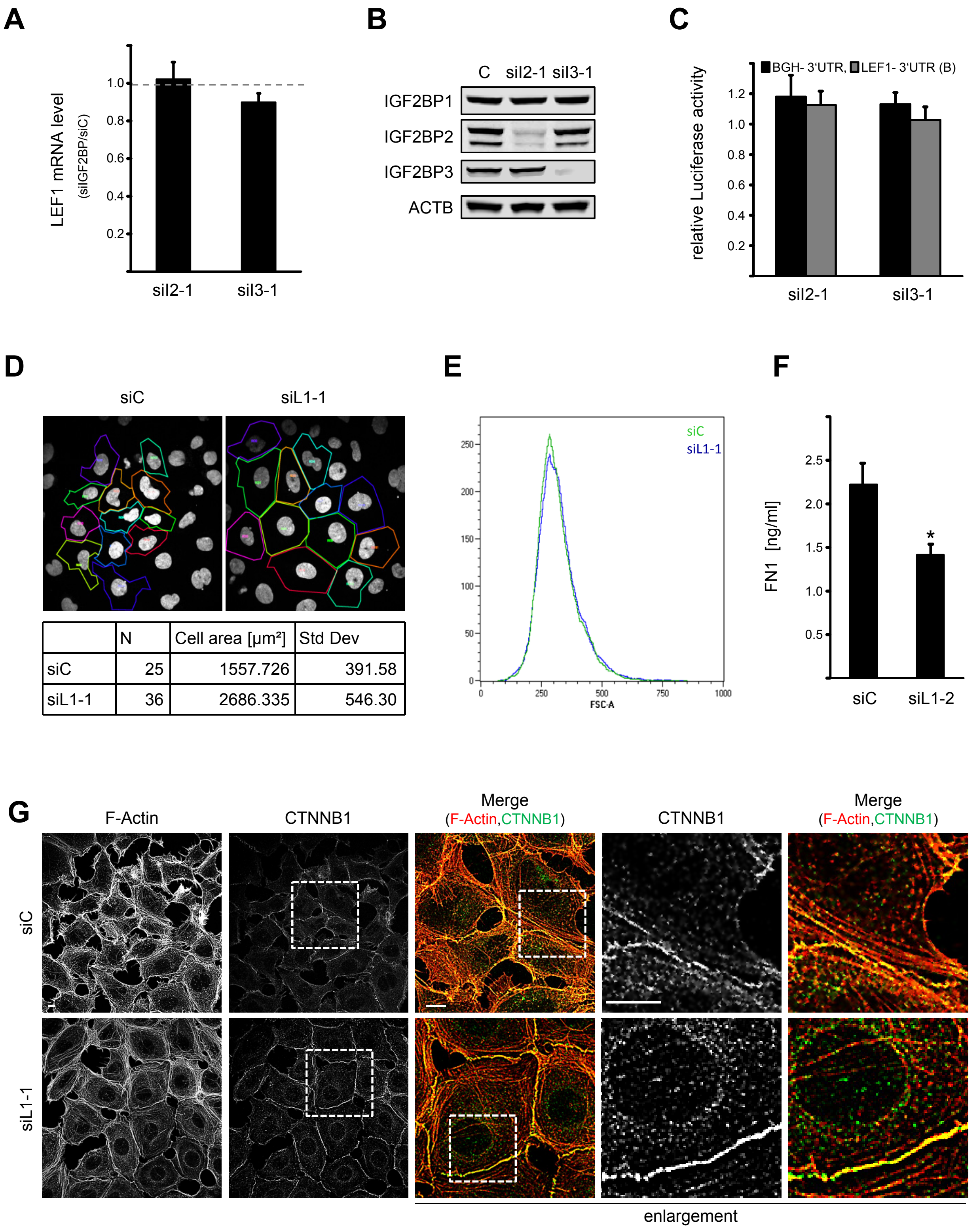

	N	Cell area [µm²]	Std Dev
siC	25	1557.726	391.58
siL1-1	36	2686.335	546.30

Figure S2, Zirkel et al.

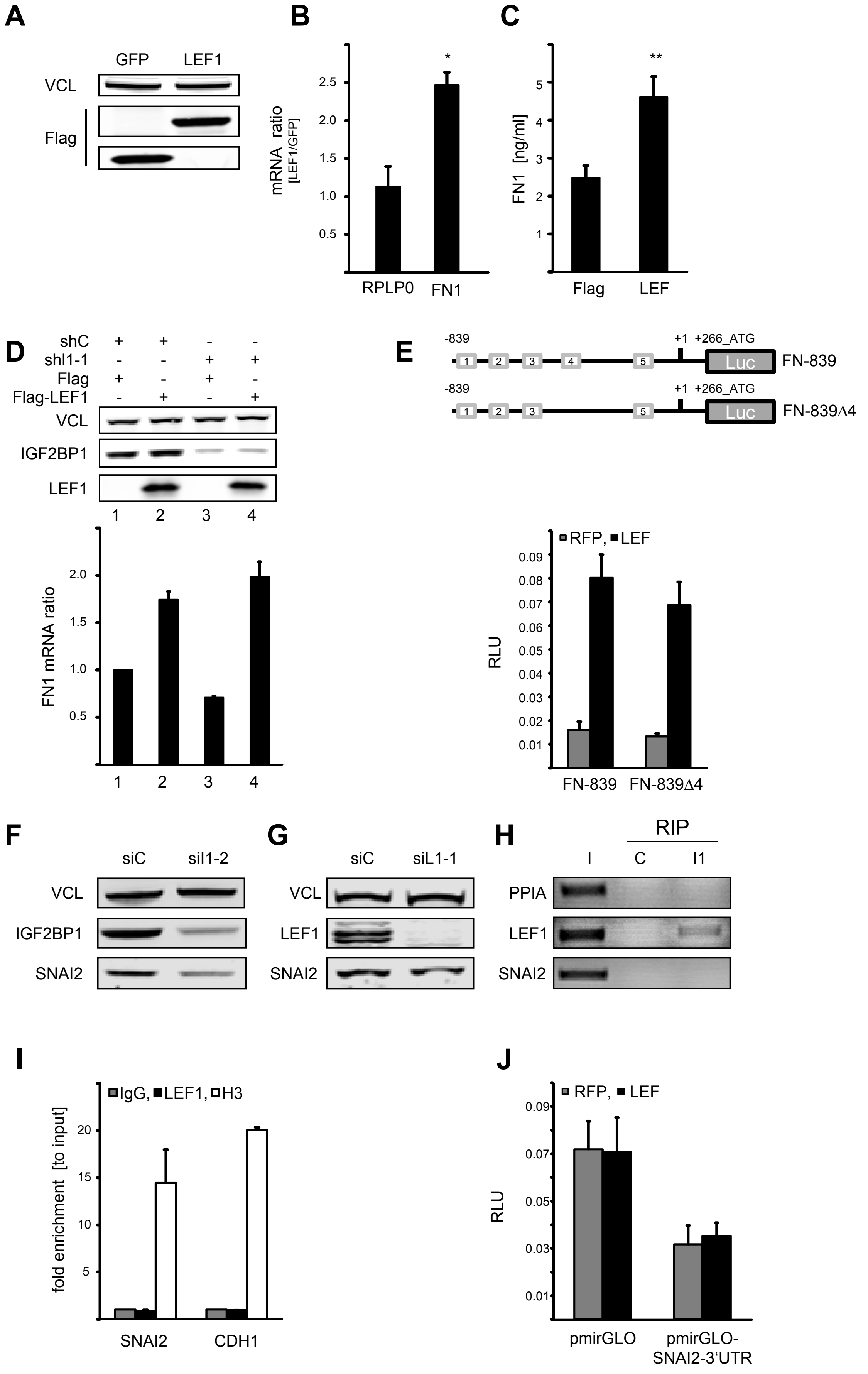

Figure S3, Zirkel et al.

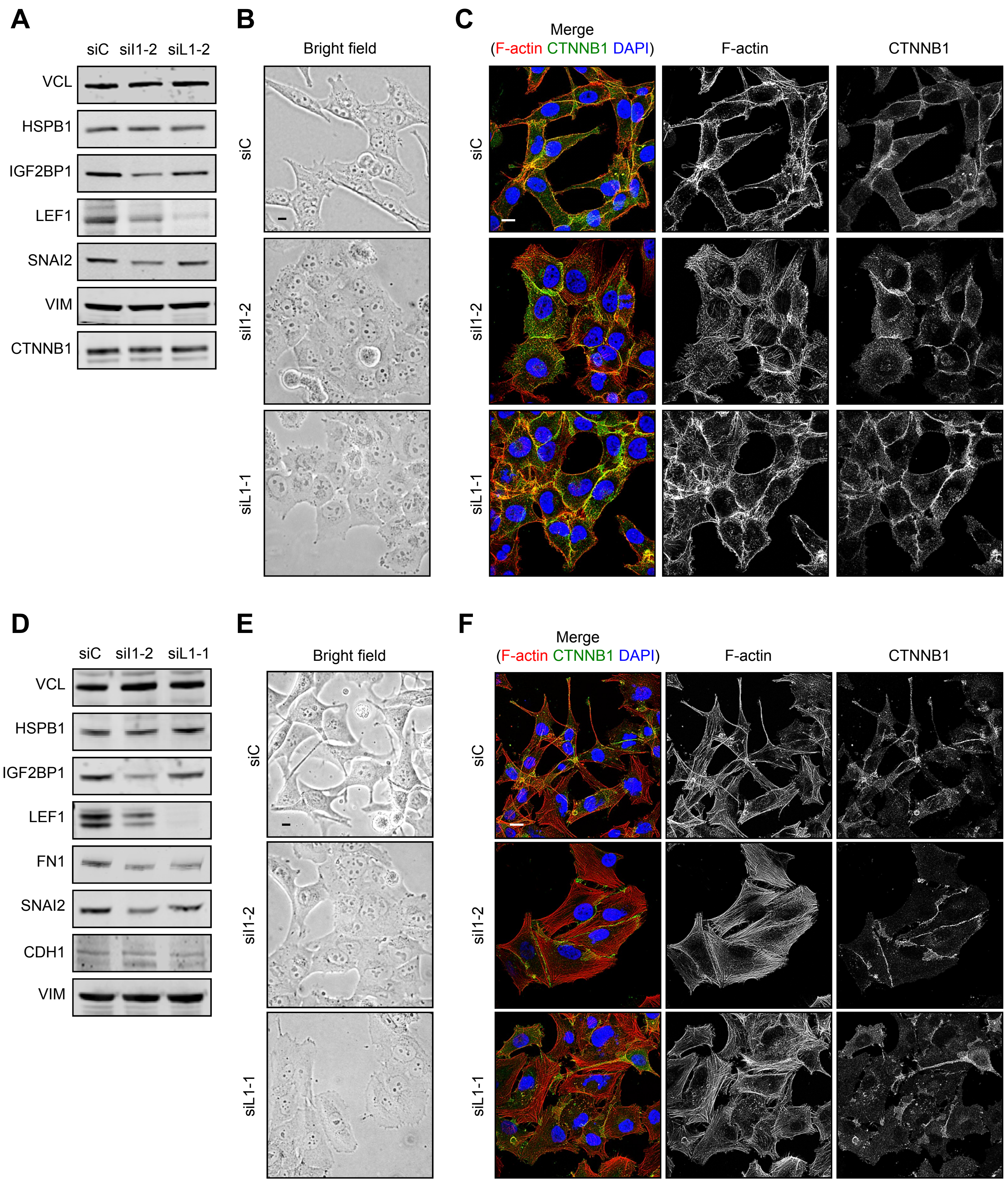

Figure S4, Zirkel et al.

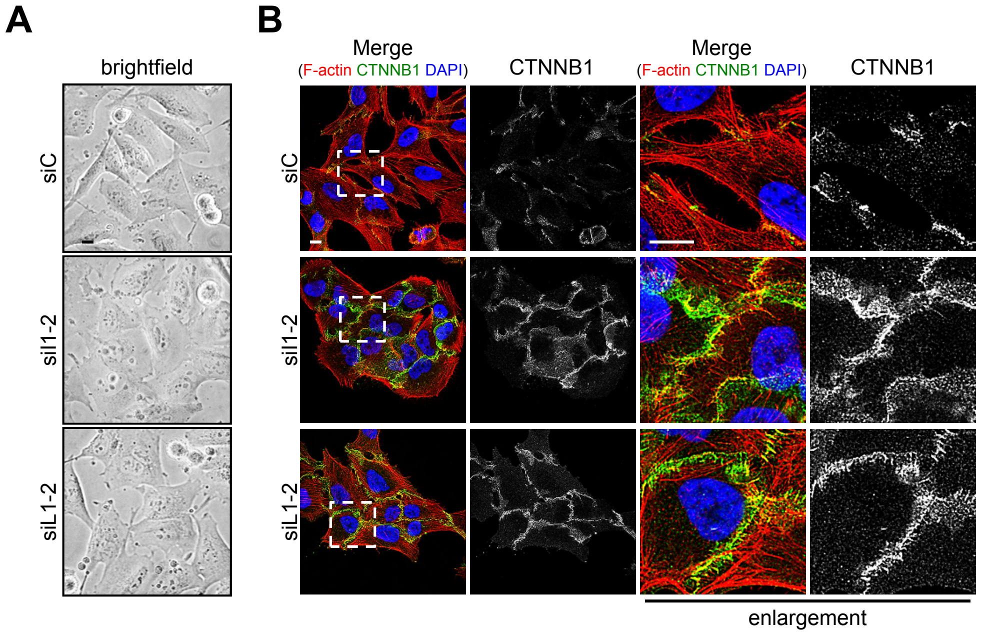

Figure S5, Zirkel et al.

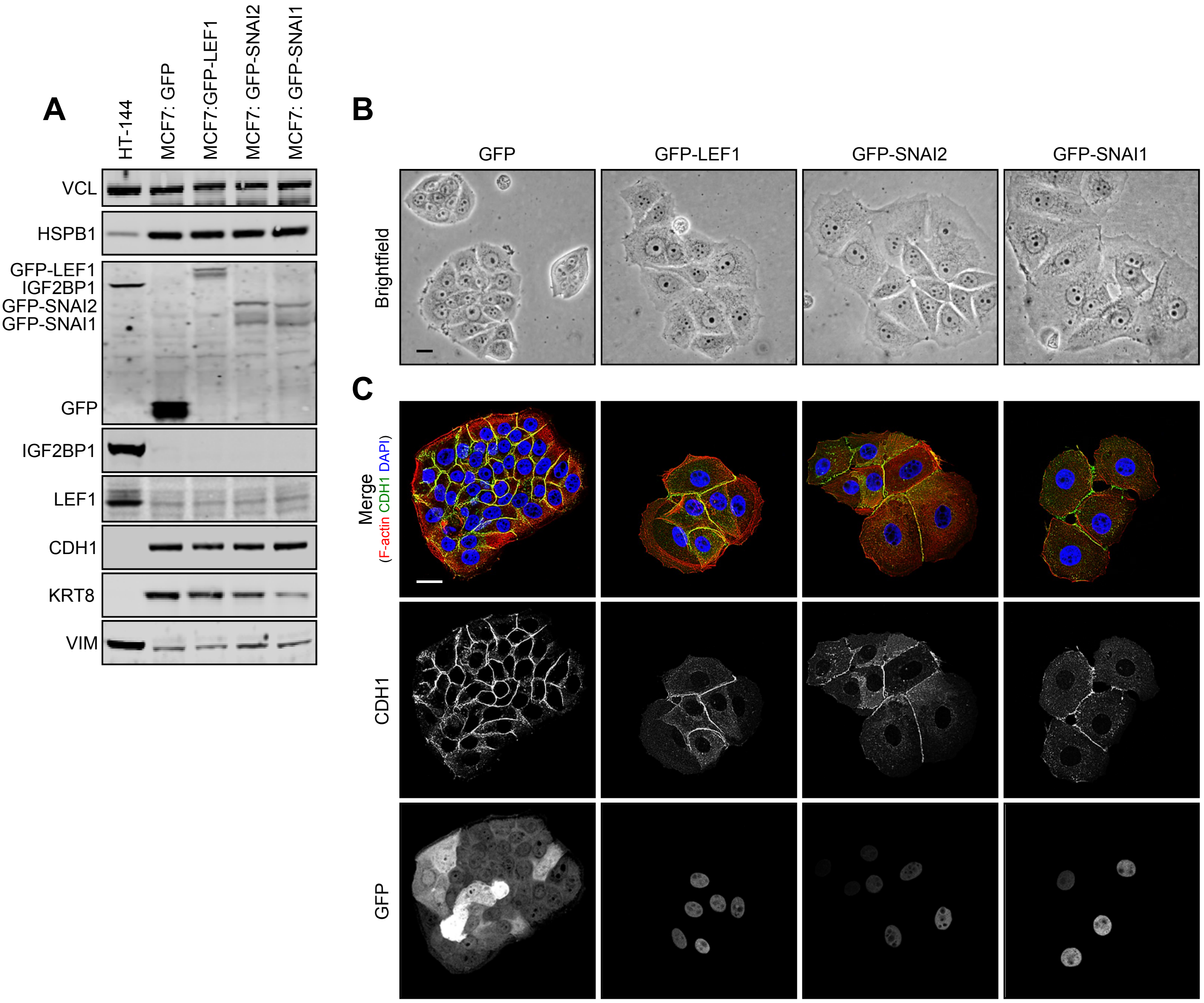

Figure S6, Zirkel et al.

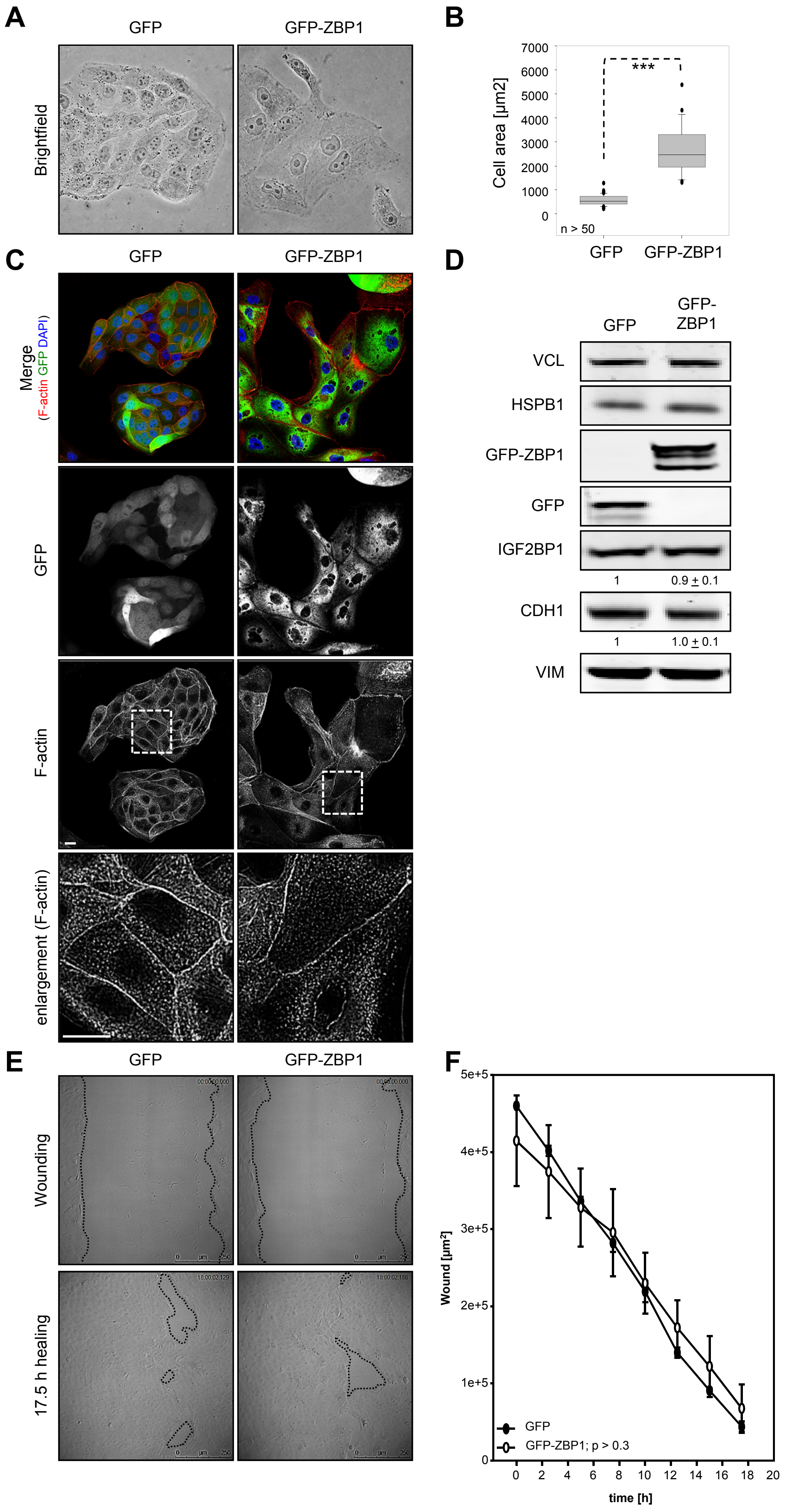

Figure S7, Zirkel et al.

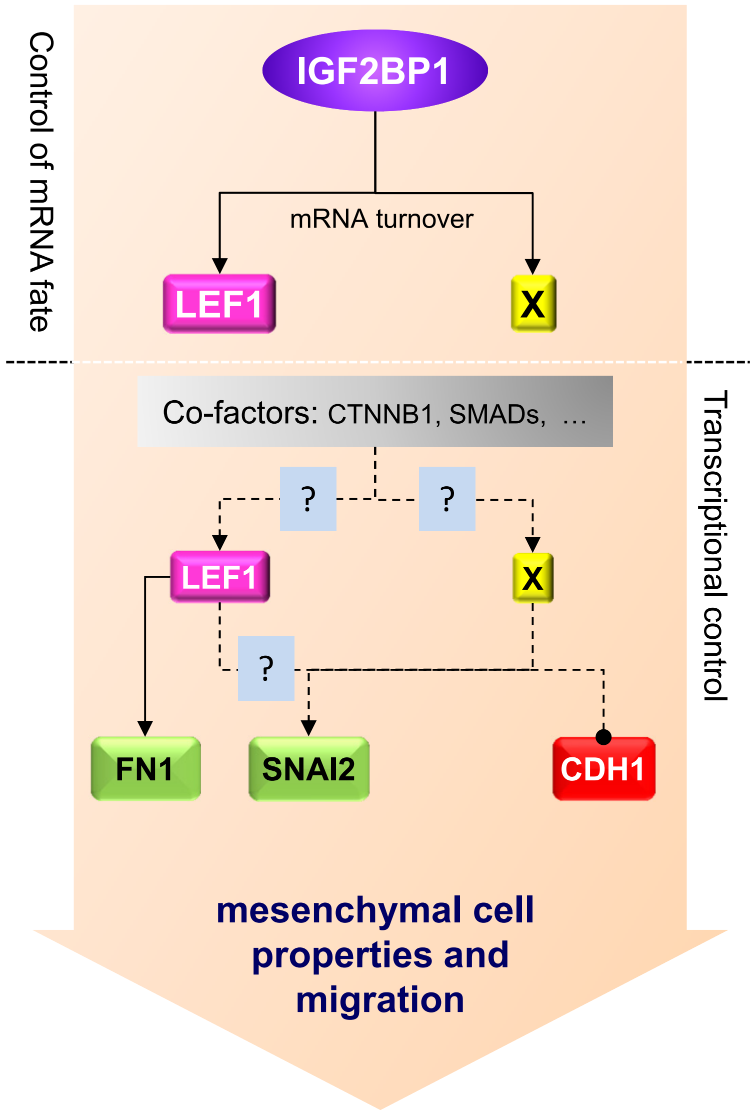

Figure S8, Zirkel et al.

Table T1: plasmids

plasmids	vector	cloning	insertion via	sense oligo (5' to 3')	antisense oligo (5' to 3')
Flag-LEF1	pcDNA3.1-Flag zeo	RT-PCR, Zero Blunt (Life Technologies)	BamHI EcoRI	ccGGATCCatgccccaactctccggaggaggtggc	ccGAATTCtcagatgtaggcagctgtcattcttggac
pLVX-LEF1	pLVX puro- new MCS	RT-PCR, Zero Blunt (Life Technologies)	BamHI EcoRI	ccGGATCCatgccccaactctccggaggaggtggc	ccGAATTCtcagatgtaggcagctgtcattcttggac
GFP-ZBP1	pEGFPC1	previously described (Huttelmaier et al., Nature 2005)			
Luc-LEF1-(A)	pcDNA3.1 FFL	RT-PCR from HEK293 cells, Zero Blunt (Life Technologies)	EcoRI XhoI	ccGAATTCaacatggtggaaaacgaagctcattcc	ccCTCGAGaaatgacaatttttaaaaatgttttattacaaagc
Luc-LEF1-(B)	pcDNA3.1 FFL	RT-PCR from HEK293 cells, Zero Blunt (Life Technologies)	EcoRI XhoI	ctGAATTCaaacccagactgtctccacggcc	ccCTCGAGaaatgacaatttttaaaaatgttttattacaaagc
Luc-SNAI2-3'UTR	pmirGLO	RT-PCR from HT-144 cells, Zero Blunt (Life Technologies)	BamHI XhoI	GGATCCGCTAGCgtgacgcaatcaatgtttactcgaacag	TCTAGACTCGAGcttgttaacaaacaattctttgtacagtgg
**promotor constructs**					
FN-839	pGL4.21	PCR from HEK293 genomic DNA, pGEMTeasy (Promega)	XhoI BglII	ccttCTCGAGaaaaagtaaactgttactttgtcc	ggAGATCTgttgagacggtgggggagag
FN-789	pGL4.21	PCR from HEK293 genomic DNA, pGEMTeasy (Promega)	XhoI BglII	ccttCTCGAGacttccccgggatctgcaaagcgcc	ggAGATCTgttgagacggtgggggagag
FN-739	pGL4.21	PCR from HEK293 genomic DNA, pGEMTeasy (Promega)	XhoI BglII	ccttCTCGAGaagctcattaaaggtctctgttc	ggAGATCTgttgagacggtgggggagag
FN-689	pGL4.21	PCR from HEK293 genomic DNA, pGEMTeasy (Promega)	XhoI BglII	ccttCTCGAGcctttctcagagccagacaggcac	ggAGATCTgttgagacggtgggggagag
FN-559	pGL4.21	PCR from HEK293 genomic DNA, pGEMTeasy (Promega)	XhoI BglII	ggCTCGAGggcagccccgccctgggactg	ggAGATCTgttgagacggtgggggagag
FN+1	pGL4.21	PCR from HEK293 genomic DNA, pGEMTeasy (Promega)	XhoI BglII	ggCTCGAGgcccgcgccggctgtgctgcac	ggAGATCTgttgagacggtgggggagag
FN-839Δ4	pGL4.21	PCRs based on FN-839 using FN-839 sense and antisense oligos and following primers; three point ligation	XhoI/EcoRV/BglII	ctGATATCagggagcgggatgggggaaaggcag	ctGATATCaagggtactgactcggactcccttat
SNAI1 promotor	pGL4.21	subcloned from Addgene; SNAI1-pGL2 (ID: 31694)	KpnI HindIII		
SNAI2 promotor	pGL4.21	PCR from HEK293 genomic DNA, pGEMTeasy (Promega); according to Lambertini et al., 2010	XhoI BamHI in BglII	ccaaCTCGAGtgtcaaaagtgtgagagaat	gggcGGATCCcttgccagcgggtctggc
**sh-plasmids**					
pLVX puro- new MCS		previously described (Stohr et al..Genes Dev. 2012)			
shC	pLVX-shRNA2	direct cloning of annealed oligos	BamHI/ EcoRI	GATCCGttgtactacacaaaagtactgTTCAAGAGAcagtacttttgtgtagtacaaTTTTTTACGCGTG	AATTCACGCGTAAAAAAttgtactacacaaaagtactgTCTCTTGAAcagtacttttgtgtagtacaaCG
shI1-1	pLVX-shRNA2	direct cloning of annealed oligos	BamHI/ EcoRI	GATCCGccgggagcagaccaggcaaTTCAAGAGAttgcctggtctgctcccggTTTTTTACGCGTG	AATTCACGCGTAAAAAAccgggagcagaccaggcaaTCTCTTGAAttgcctggtctgctcccggCG
**viral contructs**					
pLVX-shRNA2-Crimson-puro		ZsGreen casette of pLVX-shRNA2 was replaced by Crimson and puromycin resistence			
shC	pLVX-shRNA2-Crimson-puro	direct cloning of annealed oligos	BamHI/ EcoRI	GATCCGttgtactacacaaaagtactgTTCAAGAGAcagtacttttgtgtagtacaaTTTTTTACGCGTG	AATTCACGCGTAAAAAAttgtactacacaaaagtactgTCTCTTGAAcagtacttttgtgtagtacaaCG
shI1-1	pLVX-shRNA2-Crimson-puro	direct cloning of annealed oligos	BamHI/ EcoRI	GATCCGccgggagcagaccaggcaaTTCAAGAGAttgcctggtctgctcccggTTTTTTACGCGTG	AATTCACGCGTAAAAAAccgggagcagaccaggcaaTCTCTTGAAttgcctggtctgctcccggCG
shL1-1	pLVX-shRNA2-Crimson-puro	direct cloning of annealed oligos		GATCCGgaaagaaaugagagcgaauTTCAAGAGAattcgctctcatttctttcTTTTTTACGCGTG	AATTCACGCGTAAAAAAgaaagaaaugagagcgaauTCTCTTGAAattcgctctcatttctttcCG
shS2-1		Addgene; (ID: 10905)			
GFP	pLVX puro- new MCS	previously described (Stohr et al.,Genes and Development 2011)			
GFP-ZBP1	pLVX puro- new MCS	previously described (Stohr et al.,Genes and Development 2011)			
GFP-LEF1	pLVX puro- new MCS	RT-PCR, Zero Blunt (Life Technologies)	BamHI EcoRI	ccGGATCCatgccccaactctccggaggaggtggc	ccGAATTCtcagatgtaggcagctgtcattcttggac
GFP-SNAI1	pLVX puro- new MCS	PCR based on Addgene; pTK-SNAIL (ID: 36976)	EcoRI XhoI	agcGAATTCatgccgcgctccttcctggtcagg	gctCTCGAGtcagcgagggcctccggagcagcc
GFP-SNAI2	pLVX puro- new MCS	PCR based on Addgene; pTK-SLUG (ID: 36986)	EcoRI XhoI	gcaGAATTCatgccgcgctccttcctggtcaag	gcaCTCGAGtcagtgtgccacacagcagccagac

Table T2: siRNAs

siRNA	sequence (5' to 3')
siC	UUGUACUACACAAAAGUACUG
siI1-1	CCGGGAGCAGACCAGGCAA
siI1-2	UGAAUGGCCACCAGUUGGA
siI1-3	CCAUCCGCAACAUCACAAA
siI2-1	CCAUAAAGAACAUCACUAA
siI3-1	UAAGGAAGCUCAAGAUAUA
siL1-1	GAAAGAAAUGAGAGCGAAU
siL1-2	GAUGGAAGCUUGUUGAAAA

Table T4: oligos

qRT-PCR	forward (5' to 3')	reverse (5' to 3')
ACTB	AGAAAATCTGGCACCACACC	AGAGGCGTACAGGGATAGCA
CDH1	GCCGAGAGCTACACGTTCAC	GTCGAGGGAAAAATAGGCTG
CTNNB1	TCGAAATCTTGCCCTTTGTC	ATCCCGAGCTAGGATGTGAA
FN1	ACCAACCTACGGATGACTCG	GCTCATCATCTGGCCATTTT
IGF2BP1	TAGTACCAAGAGACCAGACCC	GATTTCTGCCCGTTGTTGTC
IGF2BP2	ATCGTCAGAATTATCGGGCA	GCGTTTGGTCTCATTCTGTC
IGF2BP3	AGACACCTGATGAGAATGACC	GTTTCCTGAGCCTTTACTTCC
LEF1	CGGGTACATAATGATGCCAA	TCACTGTAAGTGATGAGGGGG
MYC	AGCGACTCTGAGGAGGAAC	CGTAGTTGTGCTGATGTGTG
PPIA	GTCAACCCCACCGTGTTCTT	CTGCTGTCTTTGGGACCTTGT
RPLP0	GGCGACCTGGAAGTCCAACT	CCATCAGCACCACAGCCTTC
SNAI2	TCGGACCCACACATTACCTT	TTGGAGCAGTTTTTGCACTG
VCL	TTACAGTGGCAGAGGTGGTG	TCACGGTGTTCATCGAGTTC
**ChIP qRT-PCR**	**forward (5' to 3')**	**reverse (5' to 3')**
FN1 P1	GCGGAACTCCCGGTACTTAG	GCCTGTCTGGCTCTGAGAAA
FN1 P2	GCGCTGAGAAGGGAAGAAGT	CCATCCCGCTCCCTTTCTTT
SNAI2	TGCCCCCCTTCTCTGCCAGAGTT	TTCCGCGAAGCCAGGGGCAGCG
CDH1	TGGTGGTGTGCACCTGTACT	GGGCTTTTACACTTGGCTGA
Intergenic	CGTGCTTGTGCATTTACCCGCC	TGCCTCCATAGTGACTGCGCCT

Table T3: antibodies

primary antibody	produced in	company/provider
anti-ACTB	mouse	Sigma Aldrich
anti-CDH1	rabbit	Sigma Aldrich
anti-CDH1	rabbit	Abcam
anti-CDH2	mouse	Santa Cruz
anti-CTNNB1	rabbit	Cell Signaling
anti-CTNNB1	mouse	Santa Cruz
anti-CTNND1	mouse	BD Transductions
anti-Fibronectin	mouse	Santa Cruz
anti-Flag	mouse	Sigma Aldrich
anti-GFP	mouse	Roche
anti-HSP27 (HSPB1)	goat	Santa Cruz
anti-IGF2BP1	mouse	BSBS AB facility
anti-IGF2BP2	mouse	BSBS AB facility
anti-IGF2BP3	mouse	BSBS AB facility
anti-IgG	mouse	Millipore
anti-KRT8	rat	kind gift of AG Prof.Magin, University of Leipzig
anti-LEF1 (C18A7)	rabbit	Cell Signaling
anti-SNAI1	mouse	Cell Signaling
anti-SNAI2	rabbit	Cell Signaling
anti-TUBA4A,DM1α	mouse	Sigma Aldrich
anti-VCL	mouse	Sigma Aldrich
anti-VIM	mouse	BD Transductions
anti-ZEB1	rabbit	Santa Cruz
ChIPAb+ anti-LEF1	mouse	Millipore
**secondary antibodies**	**produced in**	**company/provider**
IRDye® 700 anti-IgG-mouse-infrared-dye	donkey	LI-COR Biosciences GmbH
IRDye® 700 anti-IgG-rabbit-infrared-dye	donkey	LI-COR Biosciences GmbH
IRDye® 800CW anti-IgG-mouse-infrared-dye	donkey	LI-COR Biosciences GmbH
IRDye® 800CW anti-IgG-rabbit-infrared-dye	donkey	LI-COR Biosciences GmbH
IRDye® 800CW anti-IgG-rat-infrared-dye	donkey	LI-COR Biosciences GmbH
IRDye® 800CW anti-IgG-goat-infrared-dye	donkey	LI-COR Biosciences GmbH
dylight488TM-conjugated anti-mouse-IgG F(ab)2	donkey	Jackson ImmunoResearch
dylight488TM-conjugated anti-rabbit IgG F(ab)2	donkey	Jackson ImmunoResearch
CyTM3-conjugated anti-mouse-IgG F(ab)2	donkey	Jackson ImmunoResearch
CyTM3-conjugated anti-rabbit-IgG F(ab)2	donkey	Jackson ImmunoResearch
dylight649TM-conjugated anti-mouse-IgG F(ab)2	donkey	Jackson ImmunoResearch
dylight649TM-conjugated anti-rabbit IgG F(ab)2	donkey	Jackson ImmunoResearch

Figure S9, Zirkel et al.
