# Supplementary material for: IGF2BP1 promotes mesenchymal cell properties and migration of tumor-derived cells by enhancing the expression of LEF1 and SNAI2 (SLUG)
Source: Nucleic Acids Res. 2013 May 15;41(13):6618–36. doi: 10.1093/nar/gkt410 (PMC3711427; doi:10.1093/nar/gkt410)
Supplement: Supplementary Data [file supp_gkt410_nar-02409-v-2012-File004.docx]

**Supplemental Figure Legends**

**Figure S1: IGF2BP1 depletion promotes epithelial cell characteristics.** **(A)** HEK293 cells were transfected with IGF2BP1-directed (siI1-2, siI1-3) or control siRNAs for 72h. Cells were fixed and processed for immunostaining of CTNNB1 and F-actin labeling by phalloidin to mark cell borders. Images were acquired by confocal microscopy. Cells were manually traced and cell size was determined by the Leica LAS/AF-software. The number of analyzed cells (N), mean cell area (µm^2^) and standard deviation of cell size (µm^2^) are indicated (lower panel). **(B)** HEK293 cells were transfected as described in (A) and harvested by tryptic digestion. Cells were subjected to flow cytometry analyses to determine relative cell volume by forward scattering using the MACSQuant (Miltenyi) software. **(C)** HEK293 cells were transfected with the indicated control, IGF2BP1-, IGF2BP2- or IGF2BP3-directed siRNAs. The mRNA levels encoding indicated IGF2BP paralogues were determined relative to controls by qRT-PCR using the ∆∆C_t_-method and cross-normalization by PPIA mRNA abundance. **(D)** Cells were transfected with two IGF2BP1-directed (siI1-1, siI1-2) or control siRNAs as in (A). Protein abundance of ACTB and IGF2BP1 was determined by Western blotting. VCL served as a loading control. Note that IGF2BP1 knockdown results in upregulated ACTB protein synthesis, as previously shown. **(E)** RNA abundance of FN1, CTNNB1 and CDH1 was determined using qRT-PCR upon IGF2BP1 depletion by indicated siRNAs as in (A). Changes in mRNA levels were determined by the ∆∆C_t_-method and cross-normalization to PPIA mRNA abundance. Statistical significance was validated by Student´s t-testing: (**) p < 0.005; (***), p < 0.0005. (**F**) HEK293 cells were transfected with indicated siRNAs as in (A). The F-actin cytoskeleton and cell-cell contact formation was analyzed by phalloidin labeling and immunostaining for CTNNB1 or CDH1. Nuclei were stained by DAPI. Images were acquired by LSM-microscopy and processed by the Leica LAS/AF-software. Cell-cell contact formation was monitored by extracting the merged signals of CTNNB1 and CDH1 using the co-localization finder software application of the Leica LAS/AF-software. Note that IGF2BP1 knockdown promotes the formation of cell-cell contact formation as indicated by increased co-localization of CDH1 and CTNNB1 at cell-cell borders.

**Figure S2**. **LEF1 promotes mesenchymal-like cell characteristics.** (**A, B**) HEK293 cells were transfected with IGF2BP2-, IGF2BP3-directed or control siRNAs for 72h. The abundance of LEF1 mRNA (A) was determined by qRT-PCR as described in Figure S1C. The selective knockdown of IGF2BP2 as well as IGF2BP3 was determined by Western blotting (B). Note that IGF2BP1 and ACTB protein amounts as well as LEF1 mRNA levels remain largely unaffected by IGF2BP2 or IGF2BP3 knockdown. (**C**) The activity of indicated luciferase reporters (also see Figure 2F) upon IGF2BP2 or IGF2BP3 knockdown was analyzed as described in Figure 2F. Note that luciferase activity remains largely unaffected by the knockdown of IGF2BP2 or IGF2BP3. These findings suggest that IGF2BP1 is the main paralogue of the IGF2BP protein family controlling LEF1 and potentially ACTB expression in HEK293 cells. **(D-G)** HEK293 cells were transfected with LEF1-directed (siL1-1) or control siRNAs for 72h. The average cell area (A) was determined as described in Figure S1A. Cell volume (E) of detached cells was analyzed by flow cytometry as described in Figure S1B. The concentration of soluble fibronectin (FN1) upon the knockdown of LEF1 (F) using an alternative siRNA (siL1-2) was determined as described in Figure 1G. Cell morphology and cell-cell contact formation was analyzed by immunostaining for CTNNB1 and F-actin by phalloidin labeling (G). Enlargements of boxed regions (left panel) are shown in the right panels (enlargement). Representative images are shown; bars, 10 µm.

**Figure S3: IGF2BP1 promotes FN1 and SNAI2 expression via LEF1. (A-C)** HEK293 cells were transfected with empty Flag-pcDNA3.1 (Flag), Flag-GFP (GFP) or Flag-LEF1 (LEF1) for 48h. The expression of LEF1 and GFP was monitored by Western blotting using anti-Flag antibody (A). VCL served as a loading control. FN1 and RPLP0 mRNA abundance in cells transfected with LEF1 was determined relative to GFP-transfected controls by qRT-PCR using the ∆∆C_t_-method and PPIA for cross-normalization (B). RPLP0 served as a control. Soluble FN1 protein concentrations were determined upon LEF1 overexpression relative to HEK293 cells transfected with pcDNA3.1-Flag by ELISA (C), as described in Figure 1G. **(D)** FN1 expression was analyzed in HEK293 cells by knockdown-recovery. Cells were co-transfected with control (shC) or IGF2BP1-directed (shI1-1) shRNAs and Flag-pcDNA3.1 or Flag-LEF1 plasmids for 72h as indicated in upper panel. IGF2BP1 knockdown and LEF1 expression were analyzed by Western blotting (upper panel). VCL served as loading control. FN1 mRNA levels were monitored by qRT-PCR using the ∆∆C_t_-method and normalization to ACTB mRNA abundance (lower panel). FN1 mRNA levels determined in controls co-transfected with empty Flag-pcDNA3.1 and control shRNAs (1) were set to one. **(E)** Schematic (upper panel) shows luciferase reporters comprising the wild type FN1 promoter sequence (FN-839) or the mutant promoter deleted by putative LEF1 binding site four (FN-839Δ4). The activity of indicated reporters (lower panel) or empty pGL4 vector was monitored in HEK293 cells upon transient co-transfection with RFP or LEF1 for 30h. Relative luciferase units (RLU) were determined by normalizing Firefly activities to Renilla activities which served as internal controls. **(F, G)** HEK293 cells were transfected with IGF2BP1-directed (siI1-2), LEF1-directed (siL1-1) or control siRNAs (siC) for 72h. The abundance of SNAI2 protein in response to IGF2BP1 (F) or LEF1 (G) knockdown was analyzed by Western blotting. VCL served as a loading control in Western blotting, as described in Figure 1F. Note that SNAI2 expression decreased significantly upon IGF2BP1 as well as LEF1 knockdown. Representative Western blots are shown. **(H)** The association of SNAI2 RNA with IGF2BP1 (I1) was examined by RIP analyses as described in Figure 2D. Co-purified RNA levels were determined by semi-quantitative PCR. IgG-beads served as control for unspecific binding (C). Note that IGF2BP1 associates with LEF1 but not SNAI2 mRNA. **(I)** Binding of endogenous LEF1 protein to the human SNAI2 or CDH1 promoters in HEK293 cells was assessed by ChIP, as described in Figure 5 D. Note, only samples for which association of LEF1 with FN1 promoter fragments (P1 and P2 in Figure 5D) could be validated were analyzed in these studies. **(J)** The relative Firefly activity of a reporter comprising the SNAI2-3’UTR was monitored in HEK293 cells, as described in (E). Note that activity of the SNAI2-3’UTR reporters is reduced compared to empty pmirGLO, serving as control. Activity, however, was not decreased by LEF1 overexpression. Error bars indicate standard deviation (s.d.) of at least three independent analyses. Statistical significance was validated by Student´s t-testing: * p < 0.05; ** p < 0.005.

**Figure S4: IGF2BP1 and LEF1 depletion impair mesenchymal-like cell morphology and marker expression in ES-2 and 1F6 cells.** **(A-C)** ES-2 ovarian carcinoma derived cells were transiently transfected with indicated siRNAs for 72h. The abundance of indicated proteins was analyzed by Western blotting using indicated antibodies (A). VCL and HSPB1 served as loading controls. Note that the knockdown of IGF2BP1 or LEF1 interfered with the expression of FN1 and SNAI2 whereas VIM or CTNNB1 expression remained largely unaffected. ES-2 cell morphology upon transfection of indicated siRNAs (72h) was monitored by bright field microscopy (B) and immunostaining of CTNNB1 and F-actin labeling by phalloidin (C). Note that the knockdown of IGF2BP1 or LEF1 interferes with mesenchymal-like cell morphology of ES-2 cells. CDH1 expression could not be monitored in ES-2 cells due to low abundance of the protein. **(D-F)** Analyses of protein abundance (D) and cell morphology (E, F) upon IGF2BP1 or LEF1 knockdown in melanoma derived 1F6 cells was performed as described in (A-C). Note that the knockdown of IGF2BP1 and LEF1 interferes with SNAI2 and FN1 expression and mesenchymal-like cell morphology whereas VIM or CDH1 abundance remains essentially unchanged. Representative images are shown; bars 10µm.

**Figure S5: IGF2BP1 and LEF1 promote the mesenchymal cell morphology in HT-144. (A, B)** Melanoma derived HT-144 cells were transiently transfected with indicated siRNAs (72h) and analyzed as described in Figure S4B, C. Enlargements of boxed regions (B, left panel) are shown in the right panels (B, enlargement). Bars, 10 µm. Note that IGF2BP1 or LEF1 knockdown interfere with mesenchymal-like cell morphology. For Western blot analyses of marker expression please refer to Figure 6B.

**Figure S6: LEF1, SNAI1 and SNAI2 induce modest morphological changes in MCF7 cells.** Epithelial-like MCF7 breast cancer derived cells were stably transduced with lentiviral vectors encoding GFP-LEF1, GFP-SNAI2 or GFP-SNAI1. **(A)** The abundance of indicated proteins was analyzed by Western blotting three weeks after transduction. HSPB1 (HSP27) and VCL served as loading controls. Mesenchymal-like HT-144 cells were used as a positive control for the expression of mesenchymal marker proteins. Note that the stable expression of the three factors only modestly affected CDH1 abundance whereas KRT8 expression was markedly reduced by SNAI1 and SNAI2. The expression of IGF2BP1 as well as LEF1 or VIM remained essentially unchanged. **(B, C)** The morphology of stably transduced MCF7 cells was analyzed by bright field microscopy (B) or upon immunostaining of CDH1 and F-actin labeling by phalloidin (C). Nuclei were stained by DAPI (C). Bars, 10 µm. Note that cell-cell contact formation remained essentially unaffected by the expression of all three factors, although it appeared modestly decreased in SNAI1 transduced cells. Strikingly, however, cell size was significantly increased by the expression of all three factors.

**Figure S7: ZBP1 overexpression promotes cell size but not mesenchymal cell morphology in MDCK cells.** Epithelial-like MDCK cells were stably transduced with lentiviral vectors encoding GFP or GFP-ZBP1. **(A-C)** The morphology of stably transduced MDCK cells was monitored by bright field (A) and fluorescence microscopy (B, C) (B). Note that cell size is significantly increased by GFP-ZBP1 overexpression. Cell-cell contacts remain largely unaffected by GFP-ZBP1 overexpression. Statistical significance was determined by Student´s t-testing: *** p < 0.0005 (n > 50). **(D)** The abundance of indicated proteins was determined by Western blotting in GFP- or GFP-ZBP1 transduced MDCK cells. VCL and HSPB1 served as loading controls. CDH1 and VIM protein abundance in GFP-ZBP1 expressing cells was determined relative to GFP controls in three independent analyses, as indicated above panels. Note that the expression of both markers remains unaffected by ZBP1. (**E, F**) Cell migration was analyzed using wound closure analyses monitored by time lapse microscopy over 18h (E; Bars, 250µm). Wound closure (area in µm^2^) of GFP-ZBP1 versus GFP expressing cell populations was quantified every 2.5h (F), essentially as described in Figure 7. Error bars indicate standard deviation of three independent scratch studies. Note that ZBP1 does not enhance cell migration compared to GFP controls.

**Figure S8: IGF2BP1 promotes mesenchymal cell properties and migration.** The scheme suggests that IGF2BP1 sustains mesenchymal-like cell properties and enhances the migratory potential of tumor-derived cells. This regulatory role involves IGF2BP1-facilitated sustainment of LEF1 and SNAI2 expression. IGF2BP1 prevents LEF1 mRNA turnover resulting in an enhancement of LEF1 expression which in turn promotes FN1 transcription by associating with the FN1 promoter. How IGF2BP1 enhances the expression of SNAI2 remains elusive (indicated by dashed lines and question mark). Our findings suggest an involvement of LEF1 in the IGF2BP1-facilitated control of SNAI2 expression but we failed to confirm association of LEF1 with the SNAI2 promoter (indicated by question mark). In addition to the EMT-driving transcriptional regulator LEF1 and SNAI2, IGF2BP1 presumably promotes or sustains the expression of additional pro-mesenchymal transcriptional regulators like ZEBs (indicated by ‘X’ and question mark), as supported by preliminary evidence (not shown). Our studies suggest that IGF2BP1 sustains pro-mesenchymal gene expression signatures by enhancing the abundance of ‘EMT-driving’ transcriptional regulators at the post-transcriptional level. How this cross-talk is modulated by transcriptional co-regulators (e.g. SMADs) remains to be addressed. IGF2BP1 appears to modulate the expression of mesenchymal (e.g. FN1) versus epithelial (e.g. CDH1) markers in a largely cell context-dependent manner. In contrast, the protein sustains mesenchymal-like cell morphology and enhances migration in all mesenchymal-like tumor-derived cells analyzed so far. The protein, however, fails to induce the expression of mesenchymal markers or significant morphological changes when expressed in epithelial-like MCF7 or MDCK cells. This suggests that IGF2BP1 is insufficient to induce EMT but rather sustains pro-mesenchymal gene expression at the post-transcriptional level.

**Figure S9: Supplemental tables summarizing material and constructs.** (T1) Plasmids and lentiviral vectors. (T2) Summary of si/shRNA sequences. (T3) Primary and secondary antibodies. (T4) List of oligonucleotides used in qRT-PCR and ChIP analyses.
